# Prevalence of left ventricular hypertrophy in children and young people with primary hypertension: Meta-analysis and meta-regression

**DOI:** 10.3389/fcvm.2022.993513

**Published:** 2022-10-31

**Authors:** Manish D. Sinha, Karolis Azukaitis, Joanna Sladowska-Kozłowska, Tonje Bårdsen, Kajus Merkevicius, Ida Sofie Karlsen Sletten, Łukasz Obrycki, Michał Pac, Fernando Fernández-Aranda, Bojko Bjelakovic, Augustina Jankauskiene, Mieczysław Litwin, HyperChildNet Working Group, Michael Hecht Olsen

**Affiliations:** ^1^Department of Paediatric Nephrology, Evelina London Children's Hospital, Guys and St Thomas' NHS Foundation Trust, London, United Kingdom; ^2^Kings College London, London, United Kingdom; ^3^Clinic of Pediatrics, Institute of Clinical Medicine, Faculty of Medicine, Vilnius University, Vilnius, Lithuania; ^4^Department of Pediatrics I, University Children's Hospital Heidelberg, Heidelberg, Germany; ^5^Department of Paediatric and Adolescent Medicine, Haukeland University Hospital, Bergen, Norway; ^6^Medical Library, University of Bergen, Bergen, Norway; ^7^Department of Nephrology, Kidney Transplantation and Hypertension, The Children's Memorial Health Institute, Warsaw, Poland; ^8^University Hospital of Bellvitge-IDIBELL, Barcelona, Spain; ^9^Department of Clinical Sciences, School of Medicine and Health Sciences, University of Barcelona, Barcelona, Spain; ^10^Clinic of Pediatrics, Clinical Center, Nis, Serbia; ^11^Medical Faculty, University of Nis, Nis, Serbia

**Keywords:** left ventricular hypertrophy, primary hypertension, children, adolescents, left ventricular mass index

## Abstract

**Background:**

Left ventricular hypertrophy (LVH) is the main marker of HMOD in children and young people (CYP). We aimed to assess the prevalence of LVH and its determinants in CYP with primary hypertension (PH).

**Methods:**

A meta-analysis of prevalence was performed. A literature search of articles reporting LVH in CYP with PH was conducted in Medline, Embase, and Cochrane databases. Studies with a primary focus on CYP (up to 21 years) with PH were included. Meta-regression was used to analyze factors explaining observed heterogeneity.

**Results:**

The search yielded a total of 2,200 articles, 153 of those underwent full-text review, and 47 reports were included. The reports evaluated 51 study cohorts including 5,622 individuals, 73% male subjects, and a mean age of 13.6 years. LVH was defined as left ventricle mass index (LVMI) ≥ 95th percentile in 22 (47%), fixed cut-off ≥38.6 g/m^2.7^ in eight (17%), sex-specific fixed cut-off values in six (13%), and miscellaneously in others. The overall prevalence of LVH was 30.5% (95% CI 27.2–33.9), while heterogeneity was high (*I*^2^ = 84%). Subgroup analysis including 1,393 individuals (76% male subjects, mean age 14.7 years) from pediatric hypertension specialty clinics and LVH defined as LVMI ≥95th percentile only (19 study cohorts from 18 studies), reported prevalence of LVH at 29.9% (95% CI 23.9 to 36.3), and high heterogeneity (*I*^2^ = 84%). Two studies involving patients identified through community screening (*n* = 1,234) reported lower LVH prevalence (21.5%). In the meta-regression, only body mass index (BMI) z-score was significantly associated with LVH prevalence (estimate 0.23, 95% CI 0.08–0.39, *p* = 0.004) and accounted for 41% of observed heterogeneity, but not age, male percentage, BMI, or waist circumference z-score. The predominant LVH phenotype was eccentric LVH in patients from specialty clinics (prevalence of 22% in seven studies with 779 participants) and one community screening study reported the predominance of concentric LVH (12%).

**Conclusion:**

Left ventricular hypertrophy is evident in at least one-fifth of children and young adults with PH and in nearly a third of those referred to specialty clinics with a predominant eccentric LVH pattern in the latter. Increased BMI is the most significant risk association for LVH in hypertensive youth.

## Introduction

Arterial hypertension (HT) is considered one of the most important global health problems and represents a potentially reversible risk factor for cardiovascular disease (CVD) development (1, 2). HT affects 4–5% of children and adolescents in general, and the prevalence increases with age (3). Importantly, childhood blood pressure (BP) has been shown to track into adulthood and is associated with subsequent cardiovascular (CV) outcomes (4). The increasing prevalence of childhood HT observed over the last decades associated with the global childhood obesity epidemic is likely to have significant implications for the development of adult CVD (5).

The heart and blood vessels are the primary organs adversely affected as a result of HT as they are directly exposed to the elevated BP, with secondary involvement of the kidneys and the central nervous system. In most cases, hypertension-mediated organ damage (HMOD) occurs in several stages. The development of diagnostic techniques makes it possible to detect early changes, which are often clinically silent and potentially reversible before established CVD (6). Persistent HT results in adaptive changes to reduce wall stress in the blood vessels and heart and include increasing thickness of blood vessels and left ventricular remodeling (7). Thus, cardiomyocyte hypertrophy and thickening of the artery wall are observed. With disease progression, the deposition of the extracellular matrix develops with an increase in the stiffness of the arterial wall and impaired function of the left ventricle and arteries (8). The development of HMOD is complex and depends on several factors including the level and duration of HT, individually variable response of tissues and organs to increased BP, and accompanying metabolic abnormalities (8–11).

Left ventricular hypertrophy (LVH) is the primary marker evaluated for HMOD. Its presence is a therapeutic indication in individuals with primary hypertension (PH) (12). Numerous studies have reported that in a substantial proportion of children and adolescents with PH, LVH is present already at diagnosis, but the extent of its prevalence has not yet been evaluated systematically. Furthermore, there are few data regarding the remodeling of the left ventricle and its determinants. The aim of our study was to estimate the prevalence of LVH and its determinants in children and young people with confirmed PH by meta-analysis and meta-regression using clearly defined inclusion and exclusion criteria and focusing on studies with PH in CYP.

## Materials and methods

A systematic review and meta-analysis to determine the prevalence of LVH and different remodeling patterns in children and young people with PH was performed. Meta-regression was used to analyze between-study factors explaining observed heterogeneity in reported prevalence.

This systematic review and meta-analysis were carried out and the data are presented according to the updated Preferred Reporting Items for Systematic Reviews and Meta-Analyses (PRISMA) guidelines (13).

### Eligibility criteria

The following eligibility criteria were used to select studies for the analysis:

#### Inclusion criteria

- Original research published since 1990.
- Studies that include children and young people (up to 21 years) with PH (or reports subgroup data for patients with PH).
- Prevalence and definition of LVH are reported.

#### Exclusion criteria

- Other languages than English.
- Reviews, case reports, case series, and animal studies.
- Studies including < 20 patients.
- Studies analyzing the data from the same or overlapping cohort.
- Studies primarily including patients with obesity or diabetes mellitus.
- Community studies where the exclusion of secondary causes of HT was not stated.
- Studies that include data from patients receiving anti-hypertensive treatment or when the treatment status of patients was not explicitly stated.

### Literature search and articles selection

A literature search was performed in MEDLINE, Embase, and Cochrane Library databases on 25 April 2022 by an academic librarian (ISKS). The search was peer-reviewed by a second academic librarian. The search included relevant synonyms and MeSH/Emtree subject headings, as well as an adapted broad search filter for children from Ovid Expert Searches to best include all pediatric and young adult studies. The literature search strategy is described in detail in the Supplementary Methods. After the removal of duplicates, all articles underwent title and abstract screening by two authors (TB and JSK). Shortlisted articles that were screened as potentially meeting eligibility criteria were independently evaluated by full-text review by at least two authors (KA, KM, TB, JSK, and MDS) and selected for final analysis. In cases where repeated data were identified between two or more studies, the study with the highest sample size or most accurate cohort description (i.e., BP phenotypes reporting) was selected.

### Definitions and data extraction

Definitions of HT, pre-hypertension (preHT), high-normal BP, and white-coat hypertension (WCH), both by office BP and by ambulatory BP monitoring (ABPM), that were proposed as per relevant international or national clinical practice guidelines were considered for the analyses. No restrictions on the method to estimate left ventricular mass index (LVMI), to define LVH and left ventricular remodeling patterns were applied.

All data extraction was performed independently by two authors and compared. The following data were extracted into pre-specified data extraction forms: first author; year of publication; description of study population based on recruitment: (i) community sample or (ii) specialty clinic; sample size and number of patients with different BP phenotypes; sex, ethnicity, mean age, and age range; mean body mass index (BMI) and waist circumference (WC) with their respective z-scores (BMIz and WCz, when available); and the number of obese individuals. For LVMI and definition of LVH, we analyzed the LVMI calculation method, definition of LVH, number of patients with LVH, severe LVH, concentric remodeling, concentric LVH, and eccentric LVH (if such data were reported). Data were extracted for the whole group of individuals with PH and per each BP phenotype (if such data were reported). In case of longitudinal studies, the data from the baseline visit were included.

If the study reported data on subgroups of different phenotypes of PH (e.g., WCH and preHT) and data were not reported for the overall/combined group or pooling was impossible, data were extracted and analyzed separately for each subgroup that were treated as independent (further referred as “study cohorts”). If a study compared several definitions of BP status and/or LVH, European Society of Hypertension (ESH) 2016 guidelines and LVH definition based on the 95th percentile cut-off were selected for primary analysis (12, 14). If absolute numbers were not indicated but could be determined by transforming data (percentage to absolute number) or confidently identified from graphs—these estimates were used.

### Critical appraisal

All studies selected for the final analysis underwent risk of bias assessment by two authors independently (JSK, TB, KM, KA) using the Joanna Briggs Institute Critical Appraisal Checklist for Studies Reporting Prevalence Data (15). Domain regarding the sufficient coverage of the sample in the analysis (number five) was not evaluated as not being relevant based on the instrument's manual. Studies scoring low risk of bias (answer “Yes”) in more than half of the evaluated domains were considered as low risk of bias. Details on the provisional guidance to use the critical appraisal tool for the present analysis are shown in Supplementary Table S1.

In cases of any discrepancies in the process of study selection, data extraction, or critical appraisal, discussions with a third reviewer led to the resolution and final decision.

### Meta-analysis and meta-regression

Descriptive characteristics of selected studies were summarized by calculating weighted means for continuous data and weighted proportions for categorical data. A meta-analysis of proportions was performed to calculate weighted pooled prevalence using a random effects model to account for expected heterogeneity. Prevalence estimates first underwent Freeman–Tukey double arcsine transformation to stabilize variance and the data were then back-transformed to provide a pooled estimate. Heterogeneity was assessed by Higgins I^2^ and Cochrane Q-tests. Mixed effects meta-regression was performed to determine between-study factors leading to observed heterogeneity. Funnel plots were created and visually inspected, as well as Egger's test was used to identify potential publication bias. All statistical analyses were performed using RStudio version 1.4.1106 and packages meta and metafor.

## Results

**Figure 1:**
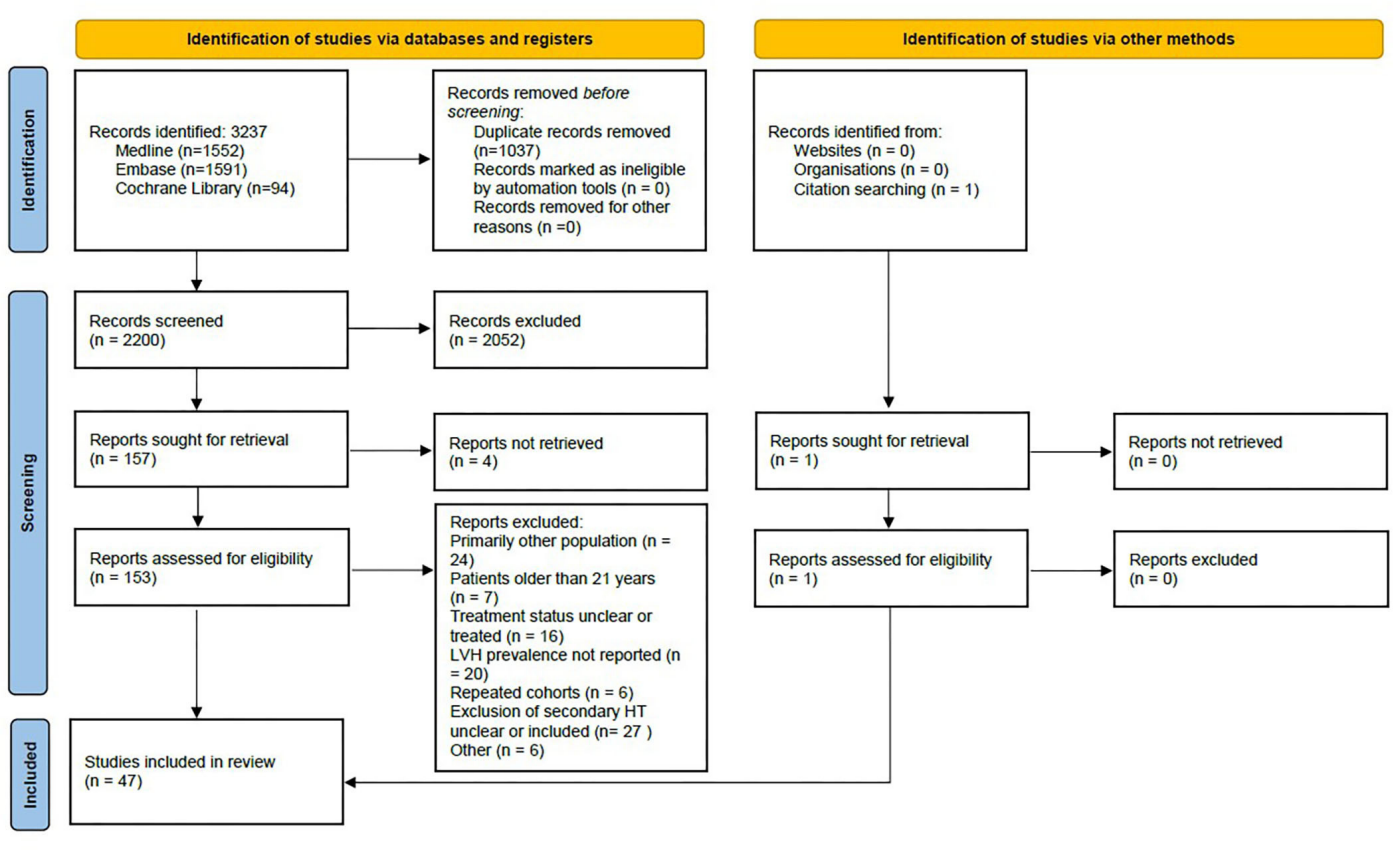
PRISMA flow diagram for article selection.

The article selection process is summarized in the PRISMA flow diagram (Figure 1). Briefly, out of 2,200 records identified through a literature search, 153 underwent full-text review (studies excluded from the analysis are shown in Supplementary Table S2) and 47 were selected for the analysis (16–62). These studies with 51 study cohorts included a total of 5,622 subjects (73% male subjects), mean age of 13.6 years (reported in 41 study cohorts), mean BMI of 25.4 kg/m^2^ (reported in 33 study cohorts), and BMIz 1.43 (21 study cohorts). The proportion of obese children (23 study cohorts) was 37%, while the mean WCz was 1.30 (8 study cohorts). Three were community screening-based cohorts (17, 30, 50), 41 reported data from pediatric HT specialty clinics, and four reported mixed data from both (31, 42, 45, 56). LVH was defined as LVMI above 95th percentile in 22 (47%) studies (17, 18, 21, 23, 25–28, 32–34, 37, 44, 46, 47, 50–53, 57, 60, 61), fixed cut-off ≥38.6 g/m^2.7^ in eight (17%) (19, 24, 36, 40, 43, 45, 49, 55), sex-specific fixed cut-offs (≥36.88 g/m^2.7^ and ≥39.36 g/m^2.7^ for female and male subjects, respectively) in six (13%) (29, 35, 38, 39, 41, 48), one used magnetic resonance imaging (MRI) (58), while the remaining were heterogeneous. Ethnicity was reported in 27 (61%) studies. Fourteen studies included subjects with WCH (28, 35, 36, 38, 45, 46, 57, 58) and/or preHT (24, 28, 37, 39, 46, 54, 57, 61). Detailed characteristics of all studies are shown in Supplementary Table S3.

### Prevalence of LVH

**Figure 2:**
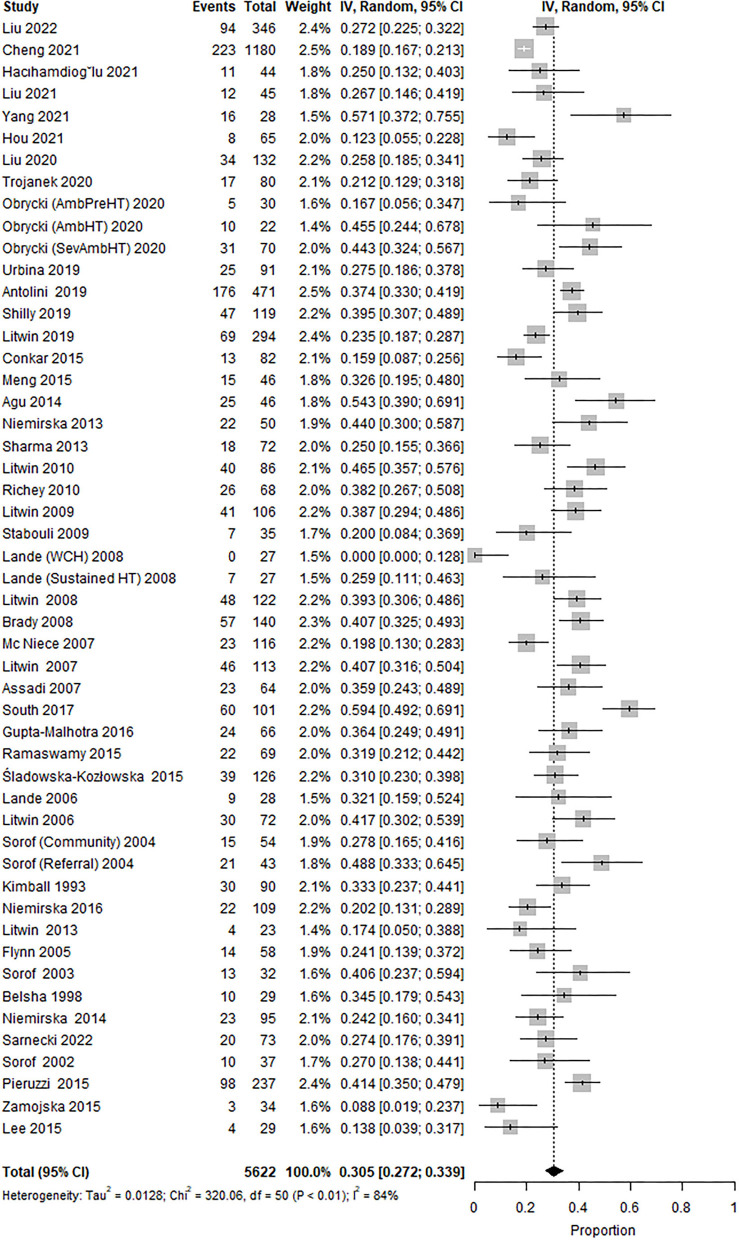
Forest plot showing the prevalence of LVH in all identified studies (47 studies, 51 study cohorts).

The overall prevalence of LVH was 30.5% (95% CI 27.2 to 33.9; Figure 2), while the heterogeneity was high (*I*^2^ = 84%).

**Figure 3:**
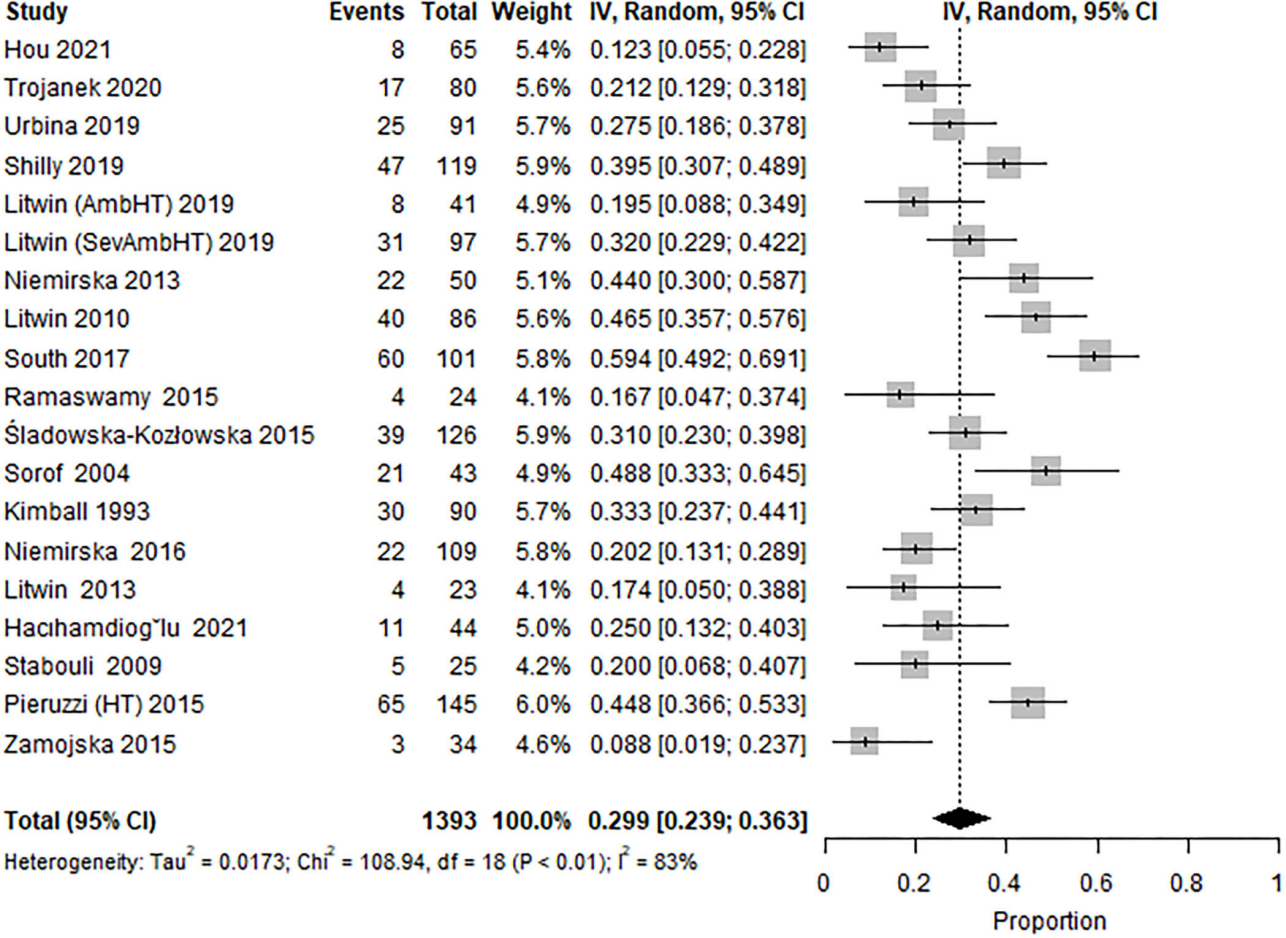
Forest plot showing the prevalence of LVH in the subset of studies from specialty clinics, LVH defined by ultrasound estimated LVMI ≥95th percentile and excluding subjects with WCH and/or preHT (18 studies, 19 study cohorts).

Two sub-group analysis studies were performed. (i) Subgroup 1: including studies reporting data from pediatric HT specialty clinics only (19 study cohorts from 18 studies) (18, 21, 23, 25, 27, 28, 32, 34, 37, 44, 46, 47, 50–53, 60, 61), reported prevalence of LVH at 29.9% (95% CI 23.9–36.3; Figure 3), and high heterogeneity (*I*^2^ = 84%). LVH in them was defined as LVMI ≥95th percentile; and excluded subjects with WCH and/or preHT and included 1,393 patients (76% male subjects) with a mean age of 14.7 years, mean BMI of 25.9 kg/m^2^ (17 study cohorts), and mean BMIz of 1.40 (14 study cohorts). Six study cohorts reported a 29% obesity rate in children, and six reported WCz (mean 1.28). The characteristics of these studies are shown in Table 1. Hypertension was defined exclusively by only office BP in six study cohorts.

**Table 1:** Characteristics of samples included in the main analysis (Subgroup 1).

**References**	**Sample population (*n*) and PH phenotype (*n*, %)**	**Ethnicity, *n* (%)**	**Age (years) Male, *n* (%)**	**BMI z-score WC z-score Obese patients, *n* (%)**	**Patients with LVH (%)**
Hacihamdioglu et al. (18)	44	NA	14.0 ± 3.19	1.33 (0.66–1.87)^c^	11 (25%)
				NA	
	NA		34 (77.3%)	26^&&^ (59.1%)	
Hou et al. (21)	65	Asian	12.4 ± 2.3	NA	8 (12.3%)
	NA		48 (73.8%)		
Trojanek et al. (23)	80	NA	15.1 ± 2	1.3 ± 0.74	17 (21.2%)
				1.5 ± 0.7	
	NA		67 (83.7%)	NA	
Urbina et al. (25)	91	White 53 (58.2%), Hispanic 15 (16.5%), Other 23 (25.3%)	15.3 ± 1.7	1.094 ±−0.81	25 (27.5%)
				NA	
	NA		54 (59.3%)	NA	
Shilly et al. (27)	119	Black 35 (29.4%), Other 84 (70.6%)	14 ± 3.3	1.2 ± 1.0	47 (39.5%)
	NA			NA	
			84 (70.6%)	33 (27.7%)	
Litwin et al. (AmbHT) (28)	41	NA	15 ± 3	0.9 ± 1.1	8 (19.5%)
	AmbHT (41, 100%)			1.1 ± 1.1	
			35 (85.4%)	NA	
Litwin et al. (SevAmbHT) (28)	97	NA	15.3 ± 2.3	1.1 ± 0.8	31 (31.9%)
	SevAmbHT (97, 100%)			0.9 ± 0.8	
			72 (74.2%)	NA	
South et al. (44)	102	White 26 (25.5%), Black 29 (28.4%), Hispanic 44 (43.1%), Other 3 (3%)	14.9* (13.1–16.3)^b^	NA	60! (59.4%)
	HT1 (48, 47%), HT2 (14, 14%)			NA	
				76^&^ (74.5%)	
			78 (76.5%)		
Niemirska et al. (52)	109	NA	15.6 ± 1.5	1.35 ± 0.83	22 (20.2%)
				1.2 ± 0.9	
	HT1 (63, 58%), HT2 (46, 42%)		90 (82.6%)	NA	
Ramaswamy et al. (46)	24	Hispanic 7 (29.2%), Caucasian 10 (41.7%), Other 7 (29.2%)	14.7 ± 3.3	1.2 ± 1.1	4 (16.7%)
				NA	
			17 (70.8%)	NA	
	NA				
Sladowska-Kozłowska et al. (47)	126	Caucasian	15*	1.75 ± 1.7	39 (30.9%)
	AmbHT (92, 73%), SevAmbHT (34, 27%)		95 (75.4%)	1.62* (−2.03 to 6.12)^*a*^	
	*NA*				
Niemirska et al. (32)	50	NA	15* (8.5–17)^a^	2.0 ± 1.5	22 (44%)
	AmbHT (21, 42%), SevAmbHT (29, 58%)				
			50 (100%)	NA	
				28 (56%)	
Litwin et al. (53)	23	NA	15.0 ± 2.1	1.16 ± 0.8	4 (17.4%)
				0.93 ± 0.9	
	NA		19 (82.6%)	NA	
Litwin et al. (34)	86	Caucasian	14.1 ± 2.4	1.8 ± 1.8	40 (46.5%)
	AmbHT (50, 58%), SevAmbHT (36, 42%)			1.8* (−1.1 to 6.1)^a^	
			66 (76.7%)	21 (24.4%)	
Stabouli et al. (37)	25	Caucasian	14.8 ± 4.2	1.28 ± 1.03	5 (20%)
	NA		13 (52%)	NA	
				NA	
Sorof et al. (50)	43	White 13 (30.2%), Hispanic 10 (23.25%), Black 18 (41.9%), Other 2 (4.65%)	13.9 ± 2.0	1.76 ± 0.76	21 (48.8%)
	NA				
				NA	
			33 (76.7%)	25^∧^ (58.1%)	
Kimball et al. (51)	90	White 46 (51.1%), Black 44 (48.9%)	14 ± 4.0	NA	30 (33.3%)
				NA^$^	
	NA		54 (60%)		
Pieruzzi et al. (61)	145	NA	NA	NA	65 (44.8%)
				NA	
	NA		84 (57.9%)	71 (48.97%)	
Zamojska et al. (60)	34	NA	15.3 ± 2.1	NA	3 (8.8%)
				NA	
	NA		27 (79.4%)		
				0	

Continuous data are represented as mean values with standard deviation, unless specified otherwise.

^*^ Median.

^a^ Data range; ^b^ Interquartile range; ^c^ 95% confidence interval.

^&^ Overweight/obesity defined as ≥85th percentile and reported together.

^&&^ Obese or overweight.

^!^ n = 101.

^∧^ In the article, the term “Overweight” was defined as BMI ≥95th percentile.

^$^ Patients with obesity were excluded in this particular study.

AmbHT, Ambulatory hypertension; BMI, body mass index; n, number of patients; HT1, hypertension stage 1; HT2, hypertension stage 2 defined according to the fourth Report (ref.). LVH, left ventricular hypertrophy. LVMI, left ventricular mass index. NA, not available or not applicable. SevAmbHT, severe ambulatory hypertension. WC, waist circumference.

(ii) Subgroup 2: additionally including study cohorts reporting data from pediatric HT specialty clinics only that defined LVH by a fixed cut-off of 38.6 g/m^2.7^. Twenty-nine study cohorts (25 studies) (18, 19, 21, 23–25, 27, 28, 32, 34, 36, 37, 40, 43, 44, 46, 47, 49–53, 55, 60, 61) were included with a total of 1,940 subjects. The estimated pooled prevalence of LVH was 31.4% and heterogeneity was similarly high (*I*^2^ = 79%) (Supplementary Figure S1). Mean age in this subset was 14.7 years, 75% were male subjects, mean BMI was 25.8 kg/m^2^ (22 study cohorts), and mean BMIz 1.38 (20 samples). The proportion of obese children and WCz was reported in 11 study cohorts and was 27% and 1.22, respectively. HT was defined by only office BP in nine study cohorts.

Severe LVH defined as LVMI >51 g/m^2.7^ was reported in 12 included studies (15 study cohorts) with 873 individuals (mean age 14.9 years, 79% male subjects), and the pooled prevalence of severe LVH was 11% (*I*^2^ = 72%) (Supplementary Figure S2) (20, 23, 24, 32, 34, 36, 39, 43, 48, 49, 59, 62).

### Left ventricle geometry

**Figure 4:**
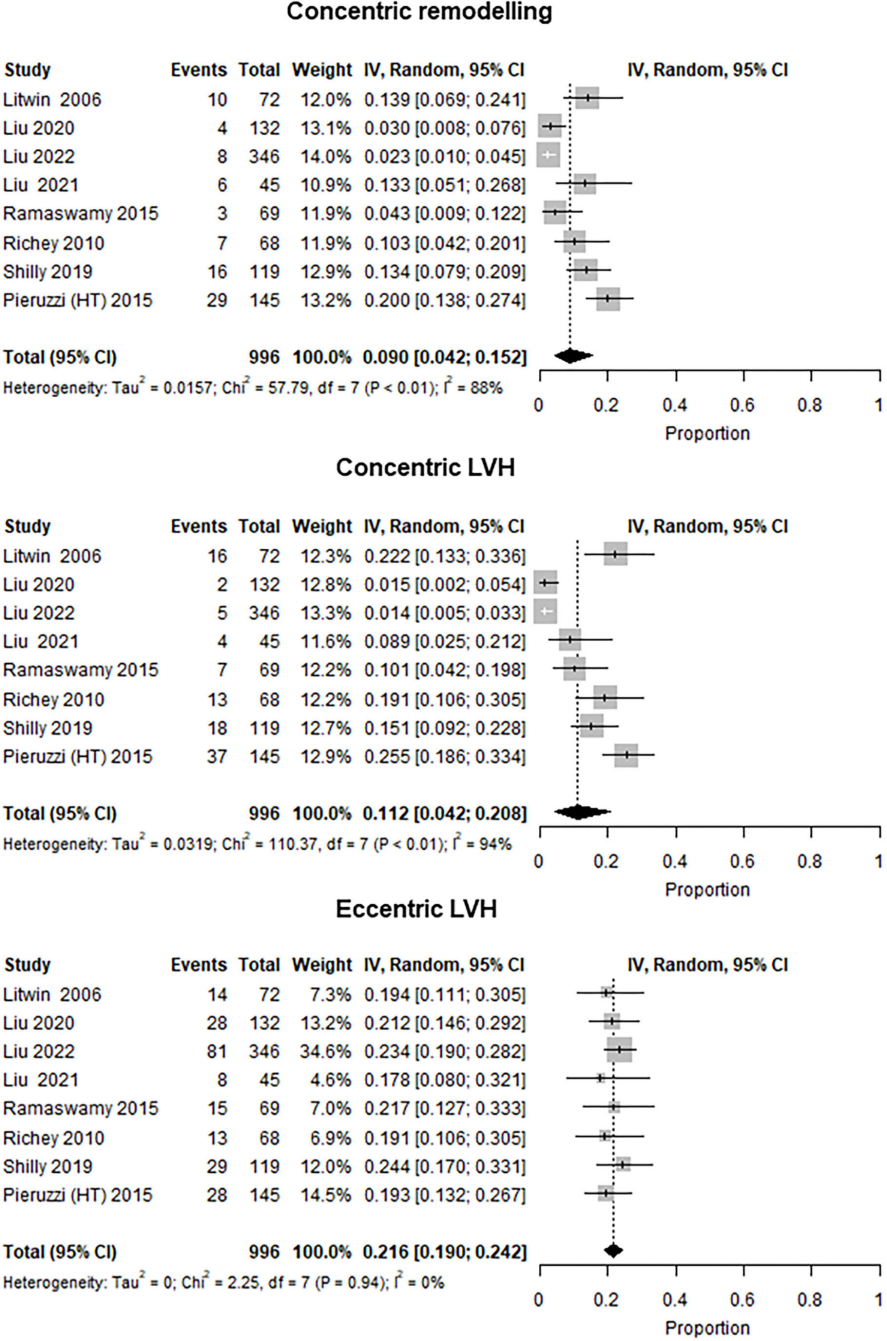
Forest plot showing the prevalence of LV geometry patterns (concentric remodeling, concentric LVH, and eccentric LVH) in studies with available data (eight studies). Three studies were excluded as being mixed [included community and specialty clinic patients: Agu et al. (31) and Gupta-Malhotra et al. (45)] or community samples (17).

Left ventricle geometry was reported in eight studies from specialty clinics that included 996 participants (mean age 13 years, 72% male subjects) with an overall prevalence of LVH at 34.2% (*I*^2^ = 70%). The prevalence of concentric remodeling was 9.0% (*I*^2^ = 88%), concentric LVH was 11.2% (*I*^2^ = 94%), and that of eccentric LVH was 21.6% (*I*^2^ = 0%) (Figure 4) (16, 19, 22, 27, 35, 46, 49, 61). In addition, one study with an overall prevalence of LVH of 18.9% reported LV geometry data for children identified by community screening (17). In this study, the predominant LV geometry pattern was concentric LVH (12%), followed by concentric remodeling (8.7%) and eccentric LVH (6.9%).

### Meta-regression

**Figure 5:**
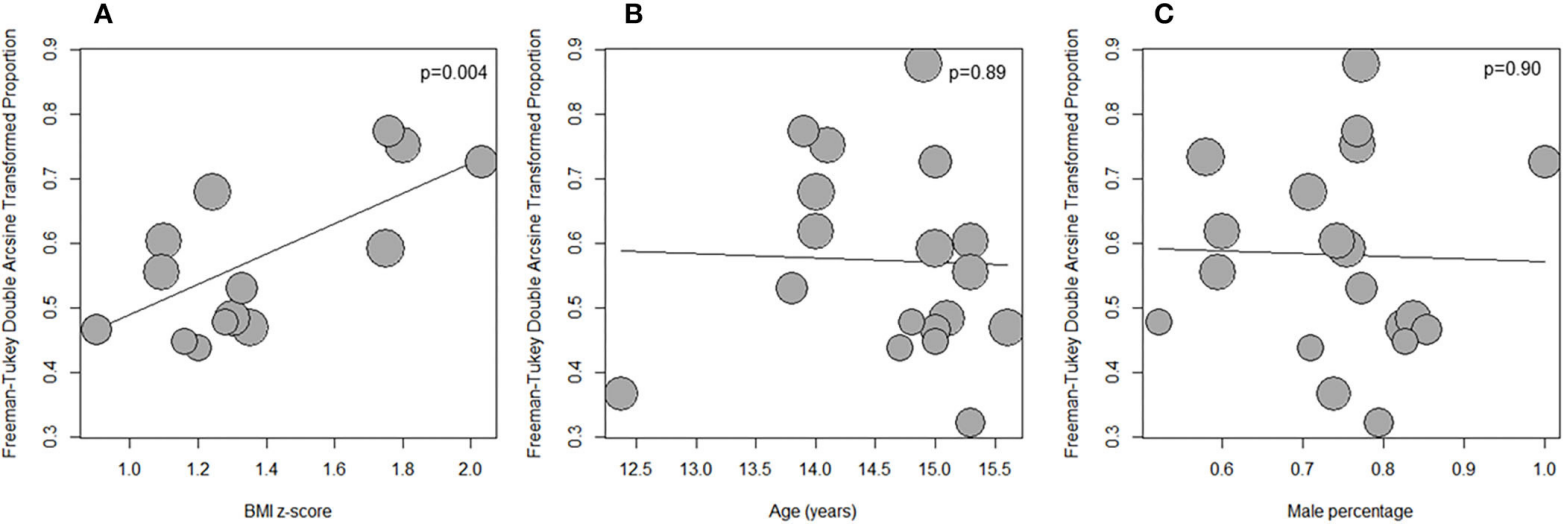
Bubble plot showing the relationship between LVH prevalence and BMI z-score **(A)**, age **(B)**, and male proportion **(C)** in the subset of studies from specialtyclinics, LVH defined by ultrasound estimated LVMI 95th percentile and excluding subjects with WCH and/or preHT.

Meta-regression for LVH prevalence was performed in Subgroup 1 with the following variables: age, proportion male, BMI, BMIz, and WCz. BMIz alone was significantly associated with LVH prevalence (estimate 0.23, 95% CI 0.08–0.39, *P* = 0.004; Figure 5A) and accounted for 41% of the observed heterogeneity. The other variables were not associated with LVH prevalence including age and sex (Supplementary Table S4; Figures 5B,C).

Repeating the analysis in Subgroup 2 revealed similar results (BMIz as explanatory variable, estimate 0.23, 95% CI 0.09–0.36, *P* = 0.001; 34.7% heterogeneity accounted for).

### Other subgroups

#### Community samples

Left ventricular hypertrophy prevalence in two community screening-based study cohorts (17, 50) was 21.5% (*I*^2^ = 60%) (Supplementary Figure S3). The studies included 1,234 participants (mean age 11.9 years, 61% male subjects).

#### White-coat and pre-hypertension

A total of 222 participants with WCH from four study cohorts were included (mean age 15.0 years, 80% male subjects) and the prevalence of LVH was 20.2% (*I*^2^ = 88%) (Supplementary Figure S4) (28, 36, 38, 46).

Three studies with preHT included 151 patients (mean age 15.2 years, 74% male subjects; ABPM used to define preHT in two studies) and the prevalence of LVH was 21.2% (*I*^2^ = 79%) (Supplementary Figure S5) (24, 28, 61).

### Risk of bias assessment

Only 19% of the studies were evaluated as low risk of bias (Supplementary Figure S6). More than half of the studies received a high risk of bias or unclear judgment on five evaluated domains: participant sampling, sample size, subject description, methods to define LVH, and reliability of LVMI measurements. Due to a small number of studies assessed as low risk of bias, the subgroup meta-analysis based on the assessed quality of the studies was not performed.

Visual inspection of Funnel plots and Egger's test suggested the risk of publication bias in Subgroups 1 and 2 (intercept 0.827, *p* = 0.07 and intercept 0.803, *p* = 0.04, respectively) with a tendency of smaller studies to report lower LVH prevalence (Supplementary Figures 7, 8).

## Discussion

The mean prevalence of LVH was estimated to be 30.5% in this systematic review and meta-analysis of 5,622 children and young people with untreated PH and 47 studies reported over the last two decades. Even with the exclusion of studies from community-based populations, studies with WCH and pre-hypertension phenotypes, and variable definitions of LVH from the analysis, the same figure of 29.9% LVH from 1,393 individuals was found. The prevalence of LVH was lower in studies at 21.5% when including children identified through community screening only.

The left ventricle is one of the primary targets for HMOD but has also been shown in hypertensive adults to be an independent risk factor for cardiovascular morbidity and mortality (63–65). Moreover, both worsening LV geometry and increasing severity of LVH are known to be associated with adverse outcomes (66–69). Although similar data associating LVH in childhood PH with subsequent CV morbidity and mortality do not exist currently, data from the Bogalusa study highlight that persistently elevated blood pressure, particularly through adolescence, is associated with a significantly higher risk for adult LVH when compared with normotensive adolescents (70). It is, therefore, widely accepted that LVH in childhood PH is an adverse surrogate marker of CV morbidity, and an important target to ameliorate future CV morbidity and mortality seen in hypertensive heart disease (12, 71).

The pathophysiology of LVH in PH is complex and not fully understood, and includes both modifiable and non-modifiable risk factors, including age, sex, ethnicity, genetic factors, and co-morbidities like obesity and metabolic syndrome (72). These observations are reflected in our study findings, which show that 76% of those with hypertension were male subjects, with a mean age of 14.6 years. Despite this, no significant relationship between age or male sex and the prevalence of LVH was found in individual studies.

In healthy children, changes in LV dimensions and mass associate with age, sex, and growth throughout childhood (14, 73–75). Increasing body size and adiposity have the highest correlation with LV mass, highlighting the increasing demands on LV (32, 39, 76–78). We observed that BMIz had a significant positive relationship with the prevalence of LVH. Other adiposity characteristics, such as the proportion of obese children or WCz were reported in fewer studies with no relationship with the prevalence of LVH following meta-analysis. The exclusion of studies primarily evaluating those with obesity, metabolic syndrome, and diabetes mellitus may also have contributed to our findings. Unfortunately, individual patient data were not available to investigate the complex interactions between BP and adiposity for the development of LVH further. BMI is the most important determinant of BP in childhood. Thus, our finding that BMI was the main determinant of LVH may be caused by the fact that increased BMI mediates the effects of other cardiovascular risk factors, such as birth weight, socioeconomic status, and metabolic abnormalities (79).

Overall, these issues highlight that the relationships among age, body size, and LVM are complex, especially during the process of growth as seen during adolescence. These complexities result in difficulties when defining LVH across the childhood age range for both sexes and are reflected in the diverse definitions for LVH by age and sex in Clinical Practice Guidelines from learned societies (12, 71). We observed that despite using different definitions of LVH reported in the literature, the prevalence of LVH remained at ~30%. These findings are in keeping with recent reports that have highlighted no differences in the prevalence of LVH when 2017 AAP vs. 2016 ESH clinical practice guidelines LVH criteria have been applied (26, 80).

One of the major criticisms of individual studies reporting the prevalence of LVH in PH is that they might reflect highly selected populations, derived from children referred to specialist centers. In keeping with this, we observed that there was a lower prevalence of LVH in studies representing a community-based population, with an estimated mean prevalence of LVH at 21.5% from 1,234 individuals. Although only two studies have reported data for children identified through community screening, this may reflect lesser severity of BP elevation or clinical presentation as opposed to those referred to specialty centers. The lower prevalence of LVH in this group highlights the importance of early identification of elevated BP to prevent HMOD development and may be in support of screening programs within the pediatric population.

As expected, those with lower levels of BP including preHT category and less severe hypertension phenotype, e.g., WCH, also had lower estimated prevalence of LVH at 21.2 and 20.2%, respectively. Although smaller in numbers, these findings are in keeping with similar observations in adults and the significant pathophysiological association of BP level with LVH (81–83).

In adult hypertensives, it has been suggested that LVH develops following a complex interaction of hemodynamic and non-hemodynamic variables (72). It has been a common view that LVH develops due to chronic pressure overload from hypertension and results in LV remodeling to reduce myocardial wall stress with the development of concentric LVH (84). Despite this traditional view, concentric LVH is not always the most common LV geometry in hypertensive adults and this may reflect other significant pathophysiological pathways and variables (85). These observations were confirmed in our study with sub-analyses with LVH prevalence of 34.2%, in whom concentric and eccentric LVH was seen in one- and two-thirds, respectively. This is perhaps unsurprising, given that LV remodeling in hypertensive adolescents reflects adaptations to increased demand on the ventricle as a result of an increase in body size, BP, but also interactions as a result of ventricular-vascular coupling at rest and after increased physical activity (86, 87). Eccentric hypertrophy is associated with obesity (88, 89), which was reflected in our study population with a mean of 25.4 kg/m^2^ and BMIz 1.43, despite the fact that these data were not reported uniformly in the studies reporting LV geometry.

In addition to cardiac remodeling, PH during childhood is associated with arterial stiffening including both structural (as assessed by carotid intima medial thickness and carotid wall cross-sectional area) and functional (as assessed by carotid-femoral pulse wave velocity) impairment of the arterial tree (24, 90). Further, although studies have previously reported LVH in the absence of other signs of arterial stiffening in childhood PH, and few investigators have performed a comprehensive evaluation of cardiac and vascular HMOD (86). We did not systematically assess arterial stiffening in subjects with LVH, but we highlight it because a better understanding of cardiac and vascular interactions during childhood in PH is required.

Based on our study findings several recommendations can be made. Despite 24-h ABPM being recommended as vital for the diagnosis of HT in this population in current guidelines (12, 71), five studies defined HT exclusively following office BP measurement. There was a large heterogeneity in the reporting of BP levels, with most studies only reporting mean values for raw data limiting the ability to analyze associations between standardized BP levels and LVH. Few studies reported ethnicity, which is a well-known predictor for BP levels (70, 77, 91, 92). Differences in the definition of LVH were common as discussed previously and can lead to significant misclassification and few studies reported LV geometry. Future studies should aim to report adjusted BP levels, BP phenotypes by ABPM, the method of indexation of LVM and its level, and the severity of LVH and LV geometry as a minimum. Future clinical practice guidelines should provide a preferred method of reporting BP levels and LVM indexation, with improved harmonization of current guidelines across ESH and AAP.

There are several limitations to this analysis, the most prominent being that none of the 47 included studies had the primary aim of reporting the prevalence of LVH in children with PH. Thus, they are unlikely to be of adequate size or optimal design to precisely estimate the prevalence of LVH in children with PH. This is in part reflected by publication bias analysis indicating lower prevalences in studies with lower sample sizes. Furthermore, other sources of biases could not have been accounted for in the original study designs. Importantly, only a few studies qualified as low risk of bias following critical appraisal, probably resulting in the observed high degree of heterogeneity between reported studies. This was mainly related to the lack of random probabilistic sampling, small sample sizes, lack of BP phenotypes descriptions, unclear duration of PH, heterogeneous LVH definitions, and lack of reporting sufficient details to confirm the reliability of LVMI measurements. In addition, different definitions of hypertension may contribute to heterogeneity in LVH prevalence, despite a recent meta-analysis finding similar predictive value between AAP and ESH guidelines (93). Finally, we were limited when evaluating by the level of BP as this was only reported in a small number of studies.

In conclusion, this systematic review and meta-analysis estimate a 30% prevalence of LVH in children and young persons with PH, highlighting the significance of childhood hypertension but also providing a treatment target for optimizing management. A large body of literature on hypertensive adults has established that LVH improves following increased physical exercise, weight loss, and anti-hypertensive therapy (94). Although similar data in hypertensive children are few and less robust, similar interventions are likely to be effective, with improved BP control following anti-hypertensive therapy and the reduction of abdominal obesity being most impactful (32, 34, 88, 95, 96). Further prospective research is required to more carefully evaluate LVH in children with PH, to understand the progression of LVH in different hypertension phenotypes and through increasing levels of BP. HMOD including cardiac and vascular assessments is needed to detect both early complications but also improve our understanding of pediatric PH.

## Data availability statement

The original contributions presented in the study are included in the article/Supplementary material, further inquiries can be directed to the corresponding author.

## Members of the HyperChildNET Working Group 3

Michael Hecht Olsen, Katerina Chrysaidou, Asle Hirth, Giacomo Simonetti, Kjell Tullus, Rina Rus, Verónica Martínez, Empar Lurbe, Elke Wuhl, Veronica Martinez Rivera.

## Author contributions

JS-K, TB, KM, MS, KA, AJ, FF-A, and ML contributed to conception and design of the study. IS performed literature search. JS-K and TB performed title and abstract screening. JS-K, TB, KM, MS, and KA performed full-text reviews, risk of bias assessment and data extraction. JS-K, KM, and TB performed data checking. KA conducted statistical analysis. MS and KA prepared the first draft of the manuscript. MS, KA, ML, TB, and JS-K wrote sections of the manuscript. JS-K, TB, KM, MS, KA, IS, AJ, BB, LO, MP, and ML contributed to critical manuscript revision, read, and approved the submitted version. All authors contributed to the article and approved the submitted version.

## Funding

This publication is based on the work of the COST Action HyperChildNET (CA19115), with the support of COST (European Cooperation in Science and Technology) and the Horizon 2020 Framework Program of the European Union.

## Conflict of interest

The authors declare that the research was conducted in the absence of any commercial or financial relationships that could be construed as a potential conflict of interest.

## Publisher's note

All claims expressed in this article are solely those of the authors and do not necessarily represent those of their affiliated organizations, or those of the publisher, the editors and the reviewers. Any product that may be evaluated in this article, or claim that may be made by its manufacturer, is not guaranteed or endorsed by the publisher.
