# Supplementary material for: Prevalence of left ventricular hypertrophy in children and young people with primary hypertension: Meta-analysis and meta-regression
Source: Front Cardiovasc Med. 2022 Oct 31;9:993513. doi: 10.3389/fcvm.2022.993513 (PMC9659762; doi:10.3389/fcvm.2022.993513)
Supplement: Supplementary file 1 [file Data_Sheet_1.PDF]

# Supplementary Material

## Prevalence of Left Ventricular Hypertrophy in Children and Young People with Primary Hypertension: Meta-analysis and Meta-regression

Manish D Sinha[1,2]*, Karolis Azukaitis[3]*, Joanna Śladowska-Kozłowska[4], Tonje Bårdsen[5], Kajus Merkevicius[3], Ida Sofie Karlsen Sletten[6], Łukasz Obrycki[7], Michał Pac[7], Fernando Fernández-Aranda[8], Bojko Bjelakovic[9], Augustina Jankauskiene[3], Mieczysław Litwin[7], on behalf of HyperChildNet Working Group 3

[1]Department of Paediatric Nephrology, Evelina London Children's Hospital, Guys & St Thomas' NHS Foundation Trust, London, UK
[2]Kings College London, London, UK
[3]Clinic of Pediatrics, Institute of Clinical Medicine, Faculty of Medicine, Vilnius University, Vilnius, Lithuania
[4]Department of Pediatrics I, University Children's Hospital Heidelberg, Heidelberg, Germany
[5]Department of Paediatric and Adolescent Medicine, Haukeland University Hospital, Bergen, Norway
[6]Medical Library, University of Bergen, Bergen, Norway.
[7]Department of Nephrology, Kidney Transplantation and Hypertension, The Children's Memorial Health Institute, Warsaw, Poland
[8]University Hospital of Bellvitge-IDIBELL and Department of Clinical Sciences, School of Medicine and Health Sciences, University of Barcelona, Spain
[9]Clinic of Pediatrics, Clinical Center, Nis, Serbia, Medical Faculty, University of Nis, Nis, Serbia

**Corresponding author:**

Dr Manish D Sinha
Email: manish.sinha@gstt.nhs.uk

**Supplemental Methods: literature search strategy**

**Database:** Ovid MEDLINE(R) and Epub Ahead of Print, In-Process, In-Data-Review & Other Non-Indexed Citations and Daily <1946 to April 22, 2022>

**Date:** 25/04/2022

1 (((left ventric* or lv) adj4 (hypertro* or mass or remodel*)) or lvh or lvmi).ti,ab,kf. 41229

2 Hypertrophy, Left Ventricular/ or Ventricular remodeling/ 27261

3 1 or 2 52587

4 hypertens*.ti,ab,kf. 482804

5 Hypertension/ or Essential Hypertension/ 249580

6 4 or 5 536160

7 (adolescen* or child* or infan* or juvenil* or minor or minors or neonat* or newborn* or new-born* or paediatric* or peadiatric* or pediatric* or perinat* or preschool* or puber* or pubescen* or school* or teen* or toddler? or youth* or student* or young adult*).ti,ab,kf. 3293210

8 adolescent/ or exp child/ or exp infant/ or young adult/ 4272716

9 7 or 8 5559612

10 3 and 6 and 9 2117

11 exp animals/ not humans/ 4997795

12 10 not 11 2003

13 limit 12 to (case reports or meta analysis or "review" or "scientific integrity review" or "systematic review") 286

14 12 not 13 1717

**15 limit 14 to yr="1990 -Current" 1552**

**Database:** Embase <1974 to 2022 April 22>

**Date:** 25/04/2022

1 (((left ventric* or lv) adj4 (hypertro* or mass or remodel*)) or lvh or lvmi).ti,ab,kf. 70333

2 heart left ventricle hypertrophy/ or heart left ventricle/ or left heart ventricle mass/ or heart ventricle remodeling/ 122448

3 1 or 2 144825

4 hypertens*.ti,ab,kf. 731662

5 hypertension/ or essential hypertension/ 682803

6 4 or 5 973500

7 (adolescen* or child* or infan* or juvenil* or minor or minors or neonat* or newborn* or new-born* or paediatric* or peadiatric* or pediatric* or perinat* or preschool* or puber* or pubescen* or school* or teen* or toddler? or youth* or student* or young adult*).ti,ab,kf. 4045415

8 exp adolescent/ or exp child/ or young adult/ 3960743

9 7 or 8 5611911

10 3 and 6 and 9 4455

11 exp animal/ not exp human/ 4936524

12 10 not 11 4113

13 limit 12 to (conference abstract or conference paper or "conference review" or "review" or short survey) 1917

14 limit 12 to (meta analysis or "systematic review") 60

15 case report/ 2732616

16 12 not (13 or 14 or 15) 1704

**17 limit 16 to yr="1990 -Current" 1591**

**Database:** Cochrane Library

**Date Run:** 25/04/2022

ID Search Hits

#1 ((("left ventric*" or lv) NEAR/3 (hypertro* or mass or remodel*)) or lvh or lvmi):ti,ab,kw 2275

#2 [mh ^"Hypertrophy, Left Ventricular"] 903

#3 [mh ^"Ventricular remodeling"] 680

#4 #1 or #2 or #3 3088

#5 (hypertens*):ti,ab,kw 70120

#6 [mh ^"Hypertension"] 18501

#7 [mh ^"Essential Hypertension"] 214

#8 #5 OR #6 OR #7 70120

#9 (adolescen* or child* or infan* or juvenil* or minor or minors or neonat* or newborn* or new-born* or paediatric* or peadiatric* or pediatric* or perinat* or preschool* or puber* or pubescen* or school* or teen* or toddler? or youth* or student* or "young adult*"):ti,ab,kw 410296

#10 [mh ^"Adolescent"] 109701

#11 [mh "Child"] 60670

#12 [mh "Infant"] 34558

#13 [mh ^"Young Adult"] 72182

#14 #9 OR #10 OR #11 OR #12 OR #13 410296

**#15 #4 AND #8 AND #14 with Cochrane Library publication date Between Jan 1990 and May 2022, in Cochrane Reviews, Cochrane Protocols, Trials, Clinical Answers, Editorials, Special Collections 94**

**Supplemental Table 1.** Guidance for the use of Joanna Briggs Institute Critical Appraisal Checklist for Studies Reporting Prevalence Data for the present meta-analysis

| **Question/Domain** | **Criteria for low risk of bias** |
|---|---|
| 1. Was the sample frame appropriate to address the target population? | • Hypertension diagnosed according to accepted (currently or at the time of publication) clinical practice guidelines<br>• Children seen consecutively in a specialty center |
| 2. Were study participants recruited in an appropriate way? | • Random probabilistic sampling from community used |
| 3. Was the sample size adequate? | • At least 322* individuals enrolled<br><br>*Estimated based on the proposed equation:<br>n= Z2P(1-P)/$d^2$ where:<br>Z=1.96 (95% confidence interval)<br>P=0.3 (expected proportion of LVH 30 %)<br>d=0.05 (precision of 5%) |
| 4. Were the study subjects and setting described in detail? | • Reported the following data: age, sex, BMI, BP levels, BP phenotypes, definitions of LVH and HT |
| 5. Was data analysis conducted with sufficient coverage of the identified sample? | • Not applicable due to lack of relevant subgroups |
| 6. Were valid methods used for the identification of the condition? | • LVH defined as LVMI above $95^{th}$ percentile |
| 7. Was the condition measured in a standard, reliable way for all participants? | • Sufficient details on the performance of imaging and its interpretation reported (e.g. who performed echocardiography, details on echocardiography protocol, review by experienced specialist, agreement if several observers) |
| 8. Was there appropriate statistical analysis? | • Number of children evaluated for LVH and with LVH clearly indicated |
| 9. Was the response rate adequate, and if not, was the low response rate managed appropriately? | • At least 70% of identified individuals with PH underwent imaging to determine LVH |

**Supplemental Table 2.** Studies excluded after full-text review and reasons for exclusion

| Excluded papers | Reason of exclusion |
|---|---|
| Kubiszewska I, Gackowska L, Obrycki L, et al. Distribution and maturation state of peripheral blood dendritic cells in children with primary hypertension. Hypertension Research - Clinical & Experimental 2022;45(3):401-13. | LVH prevalence not reported |
| Kaplinski M, Griffis H, Liu F, Tinker C, Laney NC, Mendoza M, et al. Left Ventricular Measurements and Strain in Pediatric Patients Evaluated for Systemic Hypertension and the Effect of Adequate Anti-hypertensive Treatment. Pediatr Cardiol. 2022 Jan;43(1):155–63. | Secondary HT not excluced or unclear |
| Soyaltin E, Demir BK, Erfidan G, et al. Effects of ambulatory blood pressure monitoring parameters on left ventricular mass index in hypertensive children. Blood Pressure Monitoring 2022;7:07. | LVH prevalence not reported |
| Black E, Lee J, Flynn JT, McCulloch CE, Samuels JA, Seth D, et al. Discordances between pediatric and adult thresholds in the diagnosis of hypertension in adolescents with CKD. Pediatr Nephrol. 2022 Jan;37(1):179–88. | Secondary HT not excluced or unclear |
| Zhan J, Van den Eynde J, Ozdemir E, et al. Left ventricular myocardial work indices in pediatric hypertension: correlations with conventional echocardiographic assessment and subphenotyping. European Journal of Pediatrics 2022;2:02. | Unclear treatment status |
| Abdul-Raheem JN, Binka E, Roem J, Turer CB, Urbina EM, Brady TM. Left Ventricular Diastolic Dysfunction Among Youth with Obesity and History of Elevated Blood Pressure. J Pediatr. 2021 Aug;235:130–7. | Primarily focused on obesity |
| Özden G, Kibar Gül AE, Mengen E, Ucaktürk A, Gürsu HA, Çetin İİ, et al. Investigation of the prevalence of cardiovascular risk factors in obese patients diagnosed with metabolic syndrome in childhood and examination of left ventricular function by echocardiography. J Pediatr Endocrinol Metab. 2021 Jul 27;34(7):885–96. | Primarily focused on obesity |
| Seeman T, Hradský O, Gilík J. Isolated nocturnal hypertension is associated with increased left ventricular mass index in children. Pediatr Nephrol. 2021 Jun;36(6):1543–50. | Secondary HT not excluded or unclear |
| Mercea D, Ianos R, Pop C, Lazar A-L, Sitar-Tăut A, Orăşan O, et al. The Impact of Obesity on Left Ventricular Hypertrophy and Diastolic Function in Caucasian Children. Metab Syndr Relat Disord. 2021 May;19(4):218–24. | Primarily focused on obesity |
| Gu H, Singh C, Li Y, Simpson J, Chowienczyk P, Sinha MD. Early ventricular contraction in children with primary hypertension relates to left ventricular mass. J Hypertens. 2021 Apr 1;39(4):711–7. | Includes treated patients |
| Truong G, Kamal S, Samuels JA, Bell CS. Prognostic value of ambulatory blood pressure and clinical use of echocardiography to detect left ventricular hypertrophy in children evaluated for primary hypertension. Pediatr Nephrol. 2021 Apr;36(4):961–7. | Includes treated patients |
| Merchant K, Shah PP, Singer P, Castellanos L, Sethna CB. Comparison of Pediatric and Adult Ambulatory Blood Pressure Monitoring Criteria for the Diagnosis of Hypertension and Detection of Left Ventricular Hypertrophy in Adolescents. J Pediatr. 2021 Mar;230:161–6. | Secondary HT not excluded or unclear |
| Campbell JF, Shah S, Srivaths P, Acosta AA. Reclassification of adolescent hypertension by ambulatory blood pressure monitoring using adult norms and association with left ventricular hypertrophy. J Clin Hypertens (Greenwich). 2021 Feb;23(2):265–71. | Secondary HT not excluded or unclear |
| Li Z, Duan Y, Zhao M, Magnussen CG, Xi B. Two-Year Change in Blood Pressure Status and Left Ventricular Mass Index in Chinese Children. Front Med (Lausanne). 2021;8:708044. | Secondary HT not excluded or unclear |

| | |
|---|---|
| Horlenko OM, Rusyn VI, Studenyak VM, Sochka NV, Horlenko FV, Kopolovets II, et al. Integrative morphometric characteristic of endothelial dysfunction in the cases of children with essential arterial hypertension. Wiad Lek. 2021;74(4):948–53. | Includes treated patients |
| Ozturk Z, Akcaboy M, Bideci A, et al. The association of obesity with non-dipping status and laboratory biomarkers in hypertensive children. Gazi Medical Journal 2021;32:527-31. | No definition of LVH |
| Rogowska A, Obrycki L, Kulaga Z, et al. Remodeling of Retinal Microcirculation Is Associated with Subclinical Arterial Injury in Hypertensive Children. Hypertension 2021:1203-11. | LVH prevalence not reported |
| Stambolliu E, Kollias A, Bountzona I, et al. Nighttime Home Blood Pressure in Children: Association with Ambulatory Blood Pressure and Preclinical Organ Damage. Hypertension 2021:1877-85. | LVH prevalence not reported |
| Ntineri A, Kollias A, Bountzona I, Servos G, Moyssakis I, Destounis A, et al. Twenty-four-hour ambulatory central blood pressure in adolescents and young adults: association with peripheral blood pressure and preclinical organ damage. J Hypertens. 2020 Oct;38(10):1980–8. | Out of defined age range |
| Hacıhamdioğlu DÖ, Özben Ceylan Ö , Hande A.Could arterial stiffness be early reversible target organ damage test in childhood hypertension? Anatol J Cardiol. 2021 Jul;25(7):496-504. | Repeated cohort |
| Li Y, Gu H, Sinha MD, Chowienczyk P. Hemodynamic Characterization of Primary Hypertension in Children and Adolescents. J Am Heart Assoc. 2020 Jun 16;9(12):e015097. | LVH prevalence not reported |
| Zeniodi ME, Ntineri A, Kollias A, Servos G, Moyssakis I, Destounis A, et al. Home and ambulatory blood pressure monitoring in children, adolescents and young adults: comparison, diagnostic agreement and association with preclinical organ damage. J Hypertens. 2020 Jun;38(6):1047–55. | LVH prevalence not reported |
| Tran AH, Flynn JT, Becker RC, Daniels SR, Falkner BE, Ferguson M, et al. Subclinical Systolic and Diastolic Dysfunction Is Evident in Youth With Elevated Blood Pressure. Hypertension. 2020 Jun;75(6):1551–6. | LVH prevalence not reported |
| Cilsal E. In newly diagnosed hypertensive children, increased arterial stiffness and reduced heart rate variability were associated with a non-dipping blood pressure pattern. Rev Port Cardiol (Engl Ed). 2020 Jun;39(6):331–8. | LVH prevalence not reported |
| Kawada T. Obstructive sleep apnea and left ventricular hypertrophy in obese children with hypertension. J Clin Hypertens (Greenwich). 2020 Feb;22(2):305. | LVH prevalence not reported |
| Feber J, Obrycki L, Litwin M. Prediction of left ventricular hypertrophy in children using artificial neural network. Nieren- und hochdruckkrankheiten 2020;40(3):96. | Only conference abstract |
| Yegül-Gülnar G, Kasap-Demir B, Alparslan C, Çatli G, Mutlubaş F, Yavaşcan Ö, et al. Ambulatory blood pressure monitoring parameters in obese children and adolescents with masked hypertension. Blood Press Monit. 2019 Dec;24(6):277–83. | Primarily focused on obesity |
| Di Bonito P, Licenziati MR, Di Sessa A, Manco M, Morandi A, Maffeis C, et al. A new simple formula built on the American Academy of Pediatrics criteria for the screening of hypertension in overweight/obese children. Eur J Pediatr. 2019 Aug;178(8):1291–5. | Primarily focused on obesity |
| Huang Z, Sharman JE, Fonseca R, Park C, Chaturvedi N, Davey Smith G, et al. Masked hypertension and submaximal exercise blood pressure among adolescents from the Avon Longitudinal Study of Parents and Children (ALSPAC). Scand J Med Sci Sports. 2020 Jan;30(1):25–30. | LVH prevalence not reported |
| Lang SM, Ittleman BR, Hahn E, Moore RA, Khoury PR, Ollberding NJ, et al. Comparison of Left Ventricular Mass Calculation Methods via Two-Dimensional Echocardiogram in Children, Adolescents, | Secondary HT not excluded or unclear |

| | |
|---|---|
| and Young Adults With Systemic Hypertension. Am J Cardiol. 2019 Jul 15;124(2):239–44. | |
| Hanlon CE, Binka E, Garofano JS, Sterni LM, Brady TM. The association of obstructive sleep apnea and left ventricular hypertrophy in obese and overweight children with history of elevated blood pressure. J Clin Hypertens (Greenwich). 2019 Jul;21(7):984–90. | Primarily focused on obesity |
| Di Bonito P, Valerio G, Pacifico L, Chiesa C, Invitti C, Morandi A, et al. Impact of the 2017 Blood Pressure Guidelines by the American Academy of Pediatrics in overweight/obese youth. J Hypertens. 2019 Apr;37(4):732–8. | Primarily focused on obesity |
| Lin Y, Shi L, Liu Y, Zhang H, Liu Y, Huang X, et al. Plasma Fibroblast Growth Factor 23 Is Elevated in Pediatric Primary Hypertension. Front Pediatr. 2019; 7:135. | LVH prevalence not reported |
| Floriańczyk T, Gołąbek-Dylewska M, Kucińska B, Werner B. Evaluation of left ventricular function in overweight children and teenagers with arterial hypertension and white coat hypertension. Cardiol J. 2019;26(4):343–9. | Primarily focused on obesity |
| Cilsal E, Koc AS. Renal resistive index significantly increased in hypertensive children and it is independently related to the pulse pressure and left ventricular mass index. Clin Exp Hypertens. 2019;41(7):607–14. | LVH prevalence not reported |
| Du T, Fernandez C, Barshop R, et al. Pediatric Hypertension Guidelines Improve Prediction of Adult Cardiovascular Outcomes. Hypertension 2019;73(6):1217-23. | LVH assessed in adulthood |
| Pogodina AV, Kolesnikova LR, Valyavskaya OV, et al. Periodontal health and cardiovascular risk factors in adolescents with high blood pressure. Russian Open Medical Journal 2019;8 | Unclear treatment status |
| Çelik SF, Karakurt C, Tabel Y, Elmas T, Yoloğlu S. Blood pressure is normal, but is the heart? Pediatr Nephrol. 2018 Sep;33(9):1585–91. | Includes treated patients |
| Gackowska L, Michalkiewicz J, Niemirska A, et al. Loss of CD31 receptor in CD4+ and CD8+ T-cell subsets in children with primary hypertension is associated with hypertension severity and hypertensive target organ damage. Journal of Hypertension 2018;36(11):2148-56. | LVH prevalence not reported |
| Khoury M, Khoury PR, Dolan LM, Kimball TR, Urbina EM. Clinical Implications of the Revised AAP Pediatric Hypertension Guidelines. Pediatrics. 2018 Aug;142(2). | Includes patients with diabetes |
| Schäfer M, Collins KK, Browne LP, Ivy DD, Abman S, Friesen R, et al. Effect of electrical dyssynchrony on left and right ventricular mechanics in children with pulmonary arterial hypertension. J Heart Lung Transplant. 2018 Jul;37(7):870–8. | Pulmonary hypertension |
| Yam M-C, So H-K, Kwok S-Y, Lo F-C, Mok C-F, Leung C-K, et al. Left ventricular mass of persistent masked hypertension in Hong Kong Chinese adolescents: a 4-year follow-up study. Cardiol Young. 2018 Jun;28(6):837–43. | Primarily focused on masked HT |
| Yang L, Yang L, Zhang Y, Xi B. Prevalence of Target Organ Damage in Chinese Hypertensive Children and Adolescents. Front Pediatr. 2018;6:333. | Secondary HT not excluded or unclear |
| Luo X-X, Zhu Y, Sun Y, Ge Q, Su J, So H-K, et al. Does Masked Hypertension Cause Early Left Ventricular Impairment in Youth? Front Pediatr. 2018; 6:167. | Secondary HT not excluded or unclear |
| Wójtowicz J, Łempicka A, Łuczyński W, Szczepański W, Zomerfeld A, Semeran K, et al. Central aortic pressure, arterial stiffness and echocardiographic parameters of children with overweight/obesity and arterial hypertension. Adv Clin Exp Med. 2017 Dec;26(9):1399–404. | Primarily focused on obesity |

| | |
|---|---|
| Stelcar A, Homsak E, Marcun Varda N. Assessment of Early Cardiovascular Risk in Children and Adolescents with Essential Hypertension. Klin Padiatr. 2017 Sep;229(5):286–92. | LVH prevalence not reported |
| Dibeklioglu SE, Çevik BŞ, Acar B, Özçakar ZB, Uncu N, Kara N, et al. The association between obesity, hypertension and left ventricular mass in adolescents. J Pediatr Endocrinol Metab. 2017 Feb 1;30(2):167–74. | Primarily focused on obesity |
| Navarini S, Bellsham-Revell H, Chubb H, et al. Myocardial Deformation Measured by 3-Dimensional Speckle Tracking in Children and Adolescents With Systemic Arterial Hypertension. Hypertension 2017;70(6):1142-47. | Includes treated patients |
| Supe-Markovina K, Nielsen JC, Musani M, Panesar LE, Woroniecki RP. Assessment of Left Ventricular Mass and Hypertrophy by Cardiovascular Magnetic Resonance Imaging in Pediatric Hypertension. J Clin Hypertens (Greenwich). 2016 Oct;18(10):976–81. | Secondary HT not excluded or unclear |
| Brady TM, Appel LJ, Holmes KW, Fivush B, Miller ER 3rd. Association Between Adiposity and Left Ventricular Mass in Children With Hypertension. J Clin Hypertens (Greenwich). 2016 Jul;18(7):625–33. | Treated patients included |
| Gupta-Malhotra M, Hamzeh RK, Poffenbarger T, McNiece-Redwine K, Hashmi SS. Myocardial Performance Index in Childhood Onset Essential Hypertension and White Coat Hypertension. American Journal of Hypertension. 2016;29(3):379-387 | Repeated cohort |
| Morka A, Szydlowski L, Moric-Janiszewska E, Mazurek B, Markiewicz-Loskot G, Stec S. Left Ventricular Diastolic Dysfunction Assessed by Conventional Echocardiography and Spectral Tissue Doppler Imaging in Adolescents With Arterial Hypertension. Medicine (Baltimore). 2016 Feb;95(8):e2820. | LVH as an exclusion criterion |
| Mir S, Sozeri B, Deveci M, Ozdemir K, Gun ZH, Dincel N, et al. Cardiovascular functional and structural changes in children with primary hypertension. Minerva Pediatr. 2016 Feb;68(1):27–35. | No full-text accessed |
| Johnson PK, Ferguson MA, Zachariah JP. In-Clinic Blood Pressure Prediction of Normal Ambulatory Blood Pressure Monitoring in Pediatric Hypertension Referrals. Congenital Heart Disease 2016;11(4):309-14. | Secondary HT not excluded or unclear |
| Ozturk C, Aparci M, Karaduman M, et al. Relationship of Systolic Blood Pressure and Body Mass Index With Left Ventricular Mass and Mass Index in Adolescents. Angiology 2016;67(1):58-65. | Healthy normotensive adolescents |
| Urbina EM, Khoury PR, McCoy CE, Daniels SR, Dolan LM, Kimball TR. Comparison of mercury sphygmomanometry blood pressure readings with oscillometric and central blood pressure in predicting target organ damage in youth. Blood Press Monit. 2015 Jun;20(3):150–6. | Includes patients with diabetes |
| Dušan P, Tamara I, Goran V, Gordana M-L, Amira P-A. Left ventricular mass and diastolic function in obese children and adolescents. Pediatr Nephrol. 2015 Apr;30(4):645–52. | Primarily focused on obesity |
| Bjelakovic B, Jaddoe VWV, Vukomanovic V, Lukic S, Prijic S, Krstic M, et al. The relationship between currently recommended ambulatory systolic blood pressure measures and left ventricular mass index in pediatric hypertension. Curr Hypertens Rep. 2015 Apr;17(4):534. | Secondary HT not excluded or unclear |
| Chirico D, Wade TJ, Cairney J, et al. Evidence of a hyperkinetic state in children with elevated blood pressure. Annals of Human Biology 2015;42(3):246-52. | Secondary HT not excluded or unclear |
| Dobson CP, Eide M, Nylund CM. Hypertension Prevalence, Cardiac Complications, and Antihypertensive Medication Use in Children. Journal of Pediatrics 2015;167(1):92-7.e1. | Treatment status unclear |

| | |
|---|---|
| Reschke LD, Miller ER, 3rd, Fadrowski JJ, et al. Elevated uric acid and obesity-related cardiovascular disease risk factors among hypertensive youth. Pediatric Nephrology 2015;30(12):2169-76. | Secondary HT not excluded |
| Kim HJ, Kim KH, Kil HR. Correlation between the morning hypertension on ambulatory blood pressure monitoring and the left ventricular mass in children. Korean J Pediatr. 2014 Sep;57(9):403–9. | Includes treated patients |
| Leisman D, Meyers M, Schnall J, et al. Blood pressure variability in children with primary vs secondary hypertension. Journal of Clinical Hypertension 2014;16(6):437-41. | Includes treated patients |
| Alp H, Karaarslan S, Eklioğlu BS, Atabek ME, Baysal T. The effect of hypertension and obesity on left ventricular geometry and cardiac functions in children and adolescents. J Hypertens. 2014 Jun;32(6):1283–92. | Primarily focused on obesity |
| Mirchandani D, Bhatia J, Leisman D, Kwon EN, Cooper R, Chorny N, et al. Concordance of measures of left-ventricular hypertrophy in pediatric hypertension. Pediatr Cardiol. 2014 Apr;35(4):622–6. | Secondary HT not excluded or unclear |
| Bjelakovic B, Lukic S, Vukomanovic V, Prijic S, Zivkovic N, Vasic K, et al. Blood pressure variability and left ventricular mass index in children. J Clin Hypertens (Greenwich). 2013 Dec;15(12):905–9. | Secondary HT not excluded or unclear |
| Chávez E, González E, Llanes MDC, Garí M, García Y, García Sáez J. Relationship between P wave dispersion, left ventricular mass index and blood pressure. Arch Argent Pediatr. 2013 Jun;111(3):206–12. | Not English language |
| Glowinska-Olszewska B, Tolwinska J, Luczynski W, et al. Cardiovascular risk in nonobese hypertensive adolescents: a study based on plasma biomarkers and ultrasonographic assessment of early atherosclerosis. Journal of Human Hypertension 2013;27(3):191-6. | LVH prevalence not reported |
| Pruette CS, Fivush BA, Flynn JT, Brady TM. Effects of obesity and race on left ventricular geometry in hypertensive children. Pediatr Nephrol. 2013 Oct;28(10):2015–22. | Unclear treatment status |
| Seeman T, Dostálek L, Gilík J. Control of hypertension in treated children and its association with target organ damage. Am J Hypertens. 2012 Mar;25(3):389–95. | Secondary HT not excluded or unclear |
| Sladowska-Kozlowska J, Litwin M, Niemirska A, et al. Oxidative stress in hypertensive children before and after 1 year of antihypertensive therapy. *Pediatric Nephrology*. 2012;27(10):1943-1951. | Repeated cohort |
| Sladowska-Kozłowska J, Litwin M, Niemirska A, Wierzbicka A, Wawer ZT, Janas R. Change in left ventricular geometry during antihypertensive treatment in children with primary hypertension. Pediatr Nephrol. 2011 Dec;26(12):2201–9. | Repeated cohort |
| Urbina EM, Khoury PR, McCoy C, Daniels SR, Kimball TR, Dolan LM. Cardiac and vascular consequences of pre-hypertension in youth. J Clin Hypertens (Greenwich). 2011 May;13(5):332–42. | Includes patients with diabetes |
| Dhuper S, Abdullah RA, Weichbrod L, Mahdi E, Cohen HW. Association of obesity and hypertension with left ventricular geometry and function in children and adolescents. Obesity (Silver Spring). 2011 Jan;19(1):128-33. doi: 10.1038/oby.2010.134. Epub 2010 Jun 17. PMID: 20559301. | Primarily focused on obesity |
| Stergiou GS, Giovas PP, Kollias A, et al. Relationship of home blood pressure with target-organ damage in children and adolescents. Hypertension Research - Clinical & Experimental 2011;34(5):640-4. | LVH prevalence not reported |
| Litwin M, Michałkiewicz J, Niemirska A, Gackowska L, Kubiszewska I, Wierzbicka A, et al. Inflammatory activation in children with primary hypertension. Pediatr Nephrol. 2010 Sep;25(9):1711–8. | LVH prevalence not reported |

| | |
|---|---|
| Kupferman JC, Paterno K, Mahgerefteh J, Pagala M, Golden M, Lytrivi ID, et al. Improvement of left ventricular mass with antihypertensive therapy in children with hypertension. Pediatr Nephrol. 2010 Aug;25(8):1513–8. | Secondary HT not excluded |
| Juhász M, Katona E, Settakis G, Paragh G, Molnár C, Fülesdi B, et al. Gender-related differences in adolescent hypertension and in target organ effects. J Womens Health (Larchmt). 2010 Apr;19(4):759–65. | LVH prevalence not reported |
| Páll D, Juhász M, Lengyel S, Molnár C, Paragh G, Fülesdi B, et al. Assessment of target-organ damage in adolescent white-coat and sustained hypertensives. J Hypertens. 2010 Oct;28(10):2139–44. | Unclear treatment status |
| Brady TM, Fivush B, Parekh RS, et al. Racial differences among children with primary hypertension. Pediatrics 2010;126(5):931-7. | Unclear treatment status |
| Ramaswamy P, Patel E, Fahey M, Mahgerefteh J, Lytrivi ID, Kupferman JC. Electrocardiographic predictors of left ventricular hypertrophy in pediatric hypertension. J Pediatr. 2009 Jan;154(1):106–10. | Case series |
| Maggio ABR, Aggoun Y, Marchand LM, Martin XE, Herrmann F, Beghetti M, et al. Associations among obesity, blood pressure, and left ventricular mass. J Pediatr. 2008 Apr;152(4):489–93. | Includes NT subjects |
| Assadi F. Relation of left ventricular hypertrophy to microalbuminuria and C-reactive protein in children and adolescents with essential hypertension Pediatr Cardiol 2008 May;29(3):580-4. doi: 10.1007/s00246-007-9153-4. | Repeated cohort |
| Richey PA, Disessa TG, Hastings MC, et al. Ambulatory blood pressure and increased left ventricular mass in children at risk for hypertension. Journal of Pediatrics 2008;152(3):343-8. | LVH prevalence not reported |
| Jankowski V, Meyer AA, Schlattmann P, et al. Increased uridine adenosine tetraphosphate concentrations in plasma of juvenile hypertensives. Arteriosclerosis, Thrombosis & Vascular Biology 2007;27(8):1776-81. | LVH prevalence not reported |
| Zhu H, Yan W, Ge D, Treiber FA, Harshfield GA, Kapuku G, et al. Cardiovascular characteristics in American youth with prehypertension. Am J Hypertens. 2007 Oct;20(10):1051–7. | Includes NT subjects |
| Seeman T, Gilík J, Vondrák K, Simková E, Flögelová H, Hladíková M, et al. Regression of left-ventricular hypertrophy in children and adolescents with hypertension during ramipril monotherapy. Am J Hypertens. 2007 Sep;20(9):990–6. | Secondary HT not excluded or unclear |
| Border WL, Kimball TR, Witt SA, Glascock BJ, Khoury P, Daniels SR. Diastolic filling abnormalities in children with essential hypertension. J Pediatr. 2007 May;150(5):503–9. | Treated patients included |
| Assadi F. Effect of microalbuminuria lowering on regression of left ventricular hypertrophy in children and adolescents with essential hypertension. Pediatr Cardiol. 2007 Feb;28(1):27–33. | Repeated cohort |
| Ramaswamy P, Lytrivi ID, Paul C, Golden M, Kupferman JC. Regression of left ventricular hypertrophy in children with antihypertensive therapy. Pediatr Nephrol. 2007 Jan;22(1):141–3. | Case series |
| Kavey R-EW, Kveselis DA, Atallah N, Smith FC. White coat hypertension in childhood: evidence for end-organ effect. J Pediatr. 2007 May;150(5):491–7. | Secondary HT not excluded or unclear |
| Katona E, Settakis G, Varga Z, Paragh G, Bereczki D, Fülesdi B, et al. Target-organ damage in adolescent hypertension. Analysis of potential influencing factors, especially nitric oxide and endothelin-1. J Neurol Sci. 2006 Sep 25;247(2):138–43. | LVH prevalence not reported |
| Lurbe E, Torro I, Alvarez V, Nawrot T, Paya R, Redon J, et al. Prevalence, persistence, and clinical significance of masked hypertension in youth. Hypertension. 2005 Apr;45(4):493–8. | Primarily focused on masked HT |

| | |
|---|---|
| Kowalewski M, Baszuk-Stefaniuk E, Urban M, Peczyńska J. Heart rate variability and left ventricular mass in slim children and young adults with hypertension. Kardiol Pol. 2005 Dec;63(6):605–10; discussion 611-612. | Not English language |
| Stabouli S, Kotsis V, Toumanidis S, Papamichael C, Constantopoulos A, Zakopoulos N. White-coat and masked hypertension in children: association with target-organ damage. Pediatr Nephrol. 2005 Aug;20(8):1151–5. | LVH prevalence not reported |
| Páll D, Settakis G, Katona E, Zatik J, Kollár J, Limburg M, et al. Angiotensin-converting enzyme gene polymorphism, carotid intima-media thickness, and left ventricular mass index in adolescent hypertension. J Clin Ultrasound. 2004 Apr;32(3):129–35. | LVH prevalence not reported |
| Hanevold C, Waller J, Daniels S, Portman R, Sorof J. The effects of obesity, gender, and ethnic group on left ventricular hypertrophy and geometry in hypertensive children: a collaborative study of the International Pediatric Hypertension Association. Pediatrics. 2004 Feb;113(2):328–33. | Secondary HT not excluded or unclear |
| Pall D, Settakis G, Katona E, et al. Increased common carotid artery intima media thickness in adolescent hypertension: results from the Debrecen Hypertension study. Cerebrovascular Diseases 2003;15(3):167-72. | Secondary HT not excluded or unclear |
| Daniels SR, Witt SA, Glascock B, Khoury PR, Kimball TR. Left atrial size in children with hypertension: the influence of obesity, blood pressure, and left ventricular mass. J Pediatr. 2002 Aug;141(2):186–90. | Unclear treatment status |
| Johnson MC, Bergersen LJ, Beck A, et al. Diastolic function and tachycardia in hypertensive children. American Journal of Hypertension 1999;12(10):1009-14. | Secondary HT not excluded or unclear |
| Johnson PK, Ferguson MA, Zachariah JP. In-Clinic Blood Pressure Prediction of Normal Ambulatory Blood Pressure Monitoring in Pediatric Hypertension Referrals. Congenital Heart Disease 2016;11(4):309-14. | Out of defined age range |
| Daniels SR, Loggie JM, Khoury P, Kimball TR. Left ventricular geometry and severe left ventricular hypertrophy in children and adolescents with essential hypertension. Circulation. 1998 May 19;97(19):1907-11. doi: 10.1161/01.cir.97.19.1907. PMID: 9609083. | Out of defined age range |
| de Simone G, Mureddu GF, Greco R, Scalfi L, Del Puente AE, Franzese A, et al. Relations of left ventricular geometry and function to body composition in children with high casual blood pressure. Hypertension. 1997 Sep;30(3 Pt 1):377–82. | Secondary HT not excluded |
| Chamontin B, Amar J, Barthe P, Salvador M. Blood pressure measurements and left ventricular mass in young adults with arterial hypertension screened at high school check-up. J Hum Hypertens. 1994 May;8(5):357–61. | No full-text accessed |
| Kania P, Gauthier B, Frank R, et al. A pediatric hypertension center: Two year review of an outpatient diagnostic module. Children's Hospital Quarterly 1994;6:93-97. | Patient with secondary HT not excluded |
| van Hooft IM, Grobbee DE, Waal-Manning HJ, Hofman A. Hemodynamic characteristics of the early phase of primary hypertension. The Dutch Hypertension and Offspring Study. Circulation. 1993 Apr;87(4):1100–6. | Children of hypertensive parents included |
| Hansen HS, Nielsen JR, Froberg K, Hyldebrandt N. Left ventricular hypertrophy in children from the upper five percent of the blood pressure distribution--the Odense Schoolchild Study. J Hum Hypertens. 1992 Feb;6(1):41–5. | No full-text accessed |
| Daniels SD, Meyer RA, Loggie JM. Determinants of cardiac involvement in children and adolescents with essential hypertension. Circulation. 1990 Oct;82(4):1243–8. | Out of defined age range |

| | |
|---|---|
| Daniels SR, Meyer RA, Strife CF, et al. Distribution of target-organ abnormalities by race and sex in children with essential hypertension. Journal of Human Hypertension 1990;4(2):103-4. | Out of defined age range |

## **Supplemental Table 3.** Characteristics of all selected studies

| First author, publication year | Sample | Ethnicity, n (%) | Sample population (n) | PH phenotype, n (%) | Age, years | Male, n (%) | BMI, kg/$m^2$; BMI z-score | Obese, n (%) | WC z-score | 24h SBP, mmHg; z-score | Office SBP, mmHg; z-score | 24 h DBP, mmHg; z-score | Office DBP, mmHg; z-score | HT definition | LVMI, g/$m^{2.7}$ | LVH definition | LVH, n (%) | SLVH, n (%) | Remodelling pattern, n (%) |
|---|---|---|---|---|---|---|---|---|---|---|---|---|---|---|---|---|---|---|---|
| Liu, 2022[1] | RC | Asian | 346 | Sustained HT 346 (100%) | 12.3±2.2 | 275 (79.5%) | 26.8±5.1; NA | 253 (73.1%) | NA | 126.9±10.3; NA | NA | 71.3±6.9; NA | NA | 4th Report[2] | NA | >45 g/$m^2$ | 94 (27.2%) | NA | CR 8 (2.3%), CH 5 (1.4%), EH 81 (23.4%) |
| Sarnecki, 2022[3] | RC | NA | 73 | WCH 43 (58.9%), HT 30 (41.1%) | 16.9* (15.8–17.4)[1] | 60 (82.2%) | NA[3] | 15 (20.5%) | NA | NA[3] | NA[3] | NA[3] | NA[3] | ESH, 2016[4], AHA, 2014[5] | NA[3] | 95th percentile MRI | 20 (27.4%) | NA | NA |
| Cheng, 2021[6] | CS | Asian | 1180 | NA | 11.8±3.3 | 769 (65.2%) | 23.8±5.8; NA | NA | NA | NA | 129.3±10.6; NA | NA | 72.7±7.3; NA | 4th Report[2], Flynn 2017[7] | 29.3* (24.3–36.9)[1] | 95th percentile | 223 (18.9%) | NA | CR 103 (8.7%), CH 142 (12%), EH 81 (6.9%) |
| Hacıhamdioğlu, 2021[8] | RC | NA | 44 | Sustained HT 44 (100%) | 13.8* (9.4–16.1)[2] | 34 (77.3%) | 24.1* (20.85–27.1)[1]; 1.3 (0.66–1.87)[2] | 26[&] (59.1%) | NA | 135* (130–144)[1]; NA | 138* (124–148)[1]; NA | 79* (71–87)[1]; NA | 70* (64–84)[1]; NA | Urbina, 2008[9] | 37.8±8.72 | 95th percentile | 11 (25%) | NA | NA |
| Liu, 2021[10] | RC | Asian | 45 | NA | NA[3] | 31 (68.9%) | NA[3] | 11 (24.4%) | NA | NA[3] | NA | NA[3] | NA | Fan, 2017[11] | NA[3] | ≥38.6 g/$m^{2.7}$ | 12 (26.7%) | NA | CR 6 (13.3%), CH 4 (8.9%), EH 8 (17.8%) |
| Yang, 2021[12] | RC | NA | 28 | HT1 3 (10.7%), HT2 25 (89.3%) | NA | 18 (64.3%) | 17.8±5.36; NA | NA | NA | NA | NA | NA | NA | Flynn, 2017[7] | NA | >51 g/$m^{2.7}$ | 16 (57.1%) | NA | NA |
| Hou, 2021[13] | RC | Asian | 65 | NA | 12.37±2.3 | 48 (73.8%) | 26.6±14.6; NA | NA | NA | NA | 141±11.9; NA | NA | 85.1±11.; NA | Dong, 2017[14] | 37.7±10.6 | 95th percentile | 8 (12.3%) | NA | NA |
| Liu, 2020[15] | RC | NA | 132 | HT1 67 (50.8%), HT2 65 (49.2%) | 11.9±2.1 | 103 (78%) | 26.9±5.7; NA | 98 (74.2%) | NA | NA | 125.1±9.7; NA | NA | 70.7±6.5; NA | 4th Report[2] | NA | >45 g/$m^2$ | 34 (25.8%) | NA | CR 4 (3%), CH 2 (1.5%), ER 28 (21.2%) |
| Trojanek, 2020[16] | RC | NA | 80 | NA | 15.1±2.0 | 67 (83.8%) | 26±4.2; 1.3±0.74 | NA | 84.2±9.5 (1.5±0.7) | 131±10; NA | 130±9; NA | 88±18; NA | 73±6; NA | ESH, 2016[4] | 34.3±7.6 | 95th percentile | 17 (21.3%) | 2 (2.5%) | NA |
| Obrycki, 2020[17] | RC | NA | 122 | AmbPreHT 30 (24.6%), AmbHT 22 (18%), SevAmbHT 70 (57.4%) | NA[3] | 94 (77%) | NA[3] | 34 (27.9%) | NA[3] | NA[3] | NA[3] | NA[3] | NA[3] | AHA, 2014[5] | NA[3] | ≥38.6 g/$m^{2.7}$ | 46 (37.7%) | 2 (1.6%) | NA |
| Urbina, 2019[18] | RC | White 53 (58.2%), Other 38 (41.8%) | 91 | NA | 15.3±1.7 | 54 (59.3%) | 29.5±8; 1.094±(-0.81) | NA | NA | NA | 133±7.8; NA | NA | 85.9±9.0; NA | Flynn, 2017[7] | 34.9±7.3 | 95th percentile | 9 (9.89%) | NA | NA |
| Antolini, 2019[19] | RC | NA | 471 | HN 144 (30.6%), HT 327 (69.4%) | 4–17[2] | NA | NA | 225 (47.8%) | NA | NA | NA | NA | NA | ESH, 2016[4] | NA | 95th percentile | 176 (37.4%) | NA | NA |
| Shilly, 2019[20] | RC | Black 35 (29.4%), Other 84 (70.6%) | 119 | NA | 14.3±3.3 | 84 (70.6%) | NA; 1.24±1 | 33 (27.7%) | NA | NA | NA | NA | NA | AHA, 2014[5] | 38.9±11.5 | 95th percentile | 47 (39.5%) | NA | CR 16 (13.3%), CH 18 (15.1%), EH 29 (24.4%) |
| Litwin, 2019[21] | RC | NA | 294 | WCH 127 (43.2%), PreHT 29 (9.9%), AmbHT 41 ( 13.9%), SevAmbHT 97 (33%) | 15.0±2.4 | 232 (78.9%) | NA | NA | NA | NA | NA | NA | NA | AHA, 2014[5] | NA | 95th percentile | 69 (23.5%) | NA | NA |
| South, 2017[22] | RC | White 26 (25.5%), Black 29 (28.4%), | 102 | HT1 48 (47.1%), HT2 14 (13.7%) | 14.9 (13.1–16.3)[5] | 78 (76.5%) | 28±7.9; NA | 76[&&] (74.5%) | NA | NA | 123±10; 0.89±0.91[6] | NA | 71±9 (0.4±0.77)[7] ; NA | 4th Report[2], 7th Report[2,23] | 40* (33.8–49.7)[8] | 95th percentile | 60 (59.4%)[7] | NA | NA |

| | | | | | | | | | | | | | | | | | | | |
|---|---|---|---|---|---|---|---|---|---|---|---|---|---|---|---|---|---|---|---|
| | | Hispanic 44 (43.1%), Other 3 (2.9%) | | | | | | | | | | | | | | | | | |
| Gupta-Malhotra, 2016[24] | Mixed | White 21 (31.8%), Black 23 (34.8%), Hispanic 21 (31.8%), Asian 1 (1.5%) | 66 | WCH 42 (63.6%), Sustained HT 24 (36.4%) | (10-17)[2] | 40 (60.6%) | NA | 18 (27.3%) | NA | NA | NA | NA | NA | 4th Report[2], Urbina, 2008[9] | NA | ≥38.6 $g/m^{2.7}$ | 24 (36.4%) | NA | CH 5 (7.6%), EH 19 (28.8%) |
| Niemirska, 2016[25] | RC | NA | 109 | HT1 63 (57.8%), HT2 46 (42.2%) | 15.6±1.5 | 90 (82.6%) | 26±4.2; 1.35±0.83 | NA | 1.2±0.9 | NA | 131±9; NA | NA | 76±6; NA | 4th Report[2] | 34±7.75 | 95th percentile | 22 (20.2%) | NA | NA |
| Ramaswamy, 2015[26] | RC | Hispanic 31 (44.9%), Caucasian 23 (33.3%), Other 15 (21.7%) | 69 | WCH 32 (46.4%), AmbPreHT 13 (18.8%), Sustained 24 (34.8%) | 14.8±3.3 (NA) | 52 (75.4%) | NA; 1.6±0.96 | 27 (39.1%) | NA | NA | NA | NA | NA | AHA, 2014[5] | NA | 95th percentile | 22 (31.9%) | NA | CR 3 (4.3%), CH 7 (10.1%), EH 15 (21.7%) |
| Śladowska-Kozłowska, 2015[27] | RC | Caucasian | 126 | AmbHT 92 (73%), SevAmbHT 34 (27%) | 15* (5-18)[2] | 95 (75.4%) | 24.8±4.5; 1.75 ±1.7 | NA | 1.62* (−2.03–6.12)[2] | NA | NA | NA | NA | Urbina, 2008[9] | 35.3* (19.1–62.3)[2] | 95th percentile | 39 (30.9%) | NA | NA |
| Conkar, 2015[28] | RC | NA | 82 | Sustained HT | 13.3±4.1 | 47 (57.3%) | 22.7±5.3; NA | NA | NA | NA | NA | NA | NA | Lurbe, 2009[29] | NA | ≥36.88 $g/m^{2.7}$ (female). ≥39.36 $g/m^{2.7}$ (male) | 13 (15.9%) | NA | NA |
| Meng, 2015[30] | CC | NA | 48 | NA | 13.1±0.3 | 28 (58.3%) | 26.6±0.6; NA | 32 (66.7%) | NA | NA | 131±1; NA | NA | 82±1; NA | Mi, 2010[31] | 34±5 | 95th percentile | 15[4] (32.6%) | NA | NA |
| Niemirska, 2014[32] | RC | NA | 95 | PreHT 12 (12.6%), WCH 32 (33.7%), AmbHT 7 (7.4%), SevAmbHT 44 (46.3%) | 15.3±2 | 68 (71.6%) | 25.1±3.8; 1.24±0.8 | NA | 1.2±0.8 | 129±9; NA | NA | 73±7; NA | NA | 4th Report[2], Lurbe, 2009[29] | 35±8 | 95th percentile | 23 (24.2%) | NA | NA |
| Agu, 2014[33] | Mixed | Black 15 (32.6%), White 15 (32.6%), Hispanic 15 (32.6%), Asian 1 (2.2%) | 46 | Sustained HT | 12.8±2.6 | 33 (71.7%) | 26.1±6.5; 0.915±(-0.739) | NA | NA | 126±9; NA | 136±9; NA | 70±7; NA | 79±12; NA | 4th Report[2] | 42.62±10.3 | 95th percentile | 25 (54.3%) | NA | CH 11 (23.9%), EH 14 (30.4%) |
| Litwin, 2013[34] | RC | NA | 23 | Sustained HT 23 (100%) | 15±2.1 | 19 (82.6%) | 25.2±3.8; 1.16±0.8 | NA | 1.2±0.9 | 135±8; NA | 151±9; NA | 73±7; NA | 78±9; NA | Urbina, 2008[9] | 32.5±8 | 95th percentile | 4 (17.4%) | NA | NA |
| Niemirska, 2013[35] | RC | NA | 50 | AmbHT 21 (42%), SevAmbHT 29 (58%) | 15* (8.5-17)[2] | 50 (100%) | 26.3±12.6; 2.03±1.5 | 28 (56%) | NA | 129±6; 1.6±1.1 | NA | 72±6; 0.73±1.1 | NA | 4th Report[2] | 35.5* (25.4–48.7)[2] | 95th percentile | 22 (44%) | 8 (16%) | NA |
| Sharma, 2013[36] | RC | White | 72 | NA | 12.8 (11.8–13.8)[1] | 44 (61.1%) | 23.8* (22.2–25.4)[1]; 1.16* (0.9–1.42)[1] | 27 (37.5%) | NA | 120.75* (118.10–123.39)[5]; 0.55* (0.30, 0.80)[5] | 125.57* (122.35–128.78)[5]; NA | 72.9* (70.81–74.93)[5]; -0.27* (-0.53–0.00)[5] | 71.05* (68.22–73.89)[5]; NA | Urbina, 2008[9] | NA | 95th percentile | 18 (25%) | NA | NA |

| | | | | | | | | | | | | | | | | | | | |
|---|---|---|---|---|---|---|---|---|---|---|---|---|---|---|---|---|---|---|---|
| Litwin, 2010[37] | RC | NA | 86 | AmbHT 50 (58.1%), SevAmbHT 36 (41.9%) | 14.1±2.4 | 66 (76.7%) | 25.1±4.8; 1.8±1.8 | 21 (24.4%) | NA | 130±8; NA | NA | 73±8; NA | NA | Urbina, 2008[9] | 38.5±10.7 | 95th percentile | 40 (46.5%) | 10 (11.6%) | NA |
| Richey, 2010[38] | RC | Black 50 (73.5%), Other 18 (26.5%) | 68 | WCH 16 (23.5%) | 6-18[2] | NA | NA | NA | NA | NA | NA | NA | NA | Lurbe, 2004[39] | NA | ≥36.88 $g/m^{2.7}$ (female), ≥39.36 $g/m^{2.7}$ (male) | 26 (38.2%) | NA | CR 7 (10.3%), CH 13 (19.1%), EH 13 (19.1%) |
| Litwin 2009[40] | RC | NA | 106 | Sustained HT: 70 (66%), WCH 36 (34%) | 13-17[2] | 79 (74.5%) | NA | NA | NA | NA | NA | NA | NA | 4th Report[2] | NA | ≥38.6 $g/m^{2.7}$ | 41 (38.7%) | 15 (14.2%) | NA |
| Stabouli, 2009[41] | RC | Caucasian | 35 | PreHT 10 (28.6), HT 25 (71.4%) | NA[3] | 21 (60%) | NA[3] | NA | NA | NA[3] | NA[3] | NA[3] | NA[3] | Wuhl, 2002[42] | NA[3] | 95th percentile | 7 (20%) | NA | NA |
| Lande, 2008[43] | RC | NA | 54 | WCH 27 (50%), Sustained HT 27 (50%) | NA[3] | 48 (88.9%) | NA[3] | 16 (29.6%) | NA | NA[3] | NA | NA[3] | NA | Lurbe, 2004[39] | NA[3] | ≥36.88 $g/m^{2.7}$ (female), ≥39.36 $g/m^{2.7}$ (male) | 7 (12.9%) | NA | NA |
| Litwin, 2008[44] | RC | Caucasian | 122 | PreHT 10 (8.2%), HT1 89 (72.9%), HT2 23 (18.9%) | 14.9±2.4 | 92 (75.4%) | NA | 37 (30.3%) | NA | NA | NA | NA | NA | 4th Report[2] | Not provided | ≥36.88 $g/m^{2.7}$ (female), ≥39.36 $g/m^{2.7}$ (male) | 48 (39.3%) | 14 (11.5%) | NA |
| Brady, 2008[45] | RC | White 74 (52.9%), Other 66 (47.1%) | 140 | Sustained HT | 12.9±3.6 | 88 (62.9%) | 28.9±2.1; 1.58±1 | NA | NA | NA[3] | NA | NA[3] | NA | Flynn, 2001[46] | NA | ≥36.88 $g/m^{2.7}$ (female), ≥39.36 $g/m^{2.7}$ (male) | 57 (40.7%) | NA | NA |
| McNiece, 2007[47] | Mixed | White 35 (30.2%), Black 34 (29.3%), Hispanic 47 (40.5%) | 116 | HT1 76 (65.5%), HT2 40 (34.5%) | NA[3] | 70 (60.3%) | NA | 62 (53.4%) | NA | NA | NA | NA | NA | Soergel, 1997[48] | NA | >51$g/m^{2.7}$ | 23 (19.8%) | NA | NA |
| Litwin, 2007[49] | RC | NA | 113 | Sustained HT | 14.6 (5-18)[2] | 84 (74.3%) | 24.8±4.9; 1.8±2.0 | 46 (40.7%) | NA | NA | 132±11; NA | NA | 65±8; NA | 4th Report[2] | 38.3±10.2 | ≥38.6 $g/m^{2.7}$. Severe ≥51 $g/m^{2.7}$ | 46 (40.7%) | 14 (12.4%) | NA |
| Assadi, 2007[50] | RC | Black 40 (62.5%), Hispanic 14 (21.9%), White 10 (15.6%) | 64 | HT1 36 (56.25%), HT2 28 (43.75%) | 15.3±1.7 | 33 (51.6%) | 27.3±5.1; NA | NA | NA | NA | 145±4.1; NA | NA | 87±6.5; NA | 4th Report[2] | 39.4±5.3 (NA) | ≥38.6 $g/m^{2.7}$ | 23 (35.9%) | NA | NA |
| Lande, 2006[51] | RC | Black 15 (53.6%) | 28 | HT2 3 (10.7%) | 14.9±2.3 | 22 (78.6%) | 27.7±6.1; 1.54±0.81 | 13 (46.4%) | NA | NA | 144±12; NA | NA | 77±10; NA | Soergel, 1997[48] | 36.0±7.1 | ≥36.88 $g/m^{2.7}$ (female), ≥39.36 $g/m^{2.7}$ (male) | 9 (32.1%) | 1 (3.6%) | NA |
| Litwin, 2006[52] | RC | Caucasian | 72 | Sustained HT 72 (100%) | 14.5* (5–18)[2] | 50 (69.4%) | NA | NA | NA | NA | NA | NA | NA | 4th Report[2] | NA | ≥38.6 $g/m^{2.7}$ | 30 (41.7%) | 10 (13.9%) | CR 10 (13.9%), CH 16 (22.2%), EH 14 (19.4%) |
| Flynn, 2005[53] | RC | Caucasians 67 (95.7%), Black 2 (2.9%), | 70 | PreHT 10 (14.3%), HT 60 (85.7%) | 13.3±3.9 | NA | 27.7±7.5; NA | 34 (48.6%) | NA | NA | 141±13; NA | NA | 77±12; NA | Updated task force report, 1996[54] | NA | Subjective | 14 / 58 (24.1%) | NA | NA |

| | | | | | | | | | | | | | | | | | | | |
|---|---|---|---|---|---|---|---|---|---|---|---|---|---|---|---|---|---|---|---|
| | | Asian 1 (1.4%) | | | | | | | | | | | | | | | | | |
| Sorof, 2004[55] | Mixed | White 32 (33%), Hispanic 27 (27.8%), Black 36 (37.1%), Other 2 (2.1%) | 97 | NA | NA[3] | 62 (63.9%) | NA[3] | 44 (45.4%) | NA | NA | NA[3] | NA | NA[3] | Updated task force report, 1996 [54] | NA[3] | 95th percentile | 36 (37.1%) | NA | NA |
| Sorof, 2003[56] | RC | Hispanic 15 (46.9%), Black 10 (31.25%), White 6 (18.75 %), Other 1 (3.1%) | 32 | NA | 13.9±2.7 | 25 (78.1%) | 28.2±6.8; NA | 17 (53.1%) | NA | NA | 138±10; NA | NA | 77±9; NA | Updated task force report, 1996[54] | 35.7±10.3 | ≥38.6g/$m^{2.7}$ | 13 (40.6%) | NA | NA |
| Sorof, 2002[57] | RC | White 13 (35.1%), Hispanic 12 (32.4%), Black 8 (21.6%), Asian 1 (3.1%), Other 3 (8.1) | 37 | NA | 13.5±3.7 | 28 (75.7%) | 29.1±7.1; NA | NA | NA | 126.2±13.5; NA | 138.7±14.3; NA | 72.3±7.7; NA | 77.2±8.5; NA | Updated task force report 1996[54] | 41.5±11.9 | >51g/$m^{2.7}$ | 10 (27%) | NA | NA |
| Belsha, 1998[58] | Mixed | Black 14 (48.3%) | 29 | HT1 19 (65.5%), HT2 10 (34.5%) | NA | 23 (79.3%) | NA | NA | NA | NA | NA | NA | NA | Second task force, 1987[59] | NA | >90th perc. off 32.1g/$m^3$ | 10 (34.5%) | NA | NA |
| Kimball, 1993[60] | RC | White 46 (51.1%), Black 44 (48.9%) | 90 | NA | 14±4 | 54 (60%) | 24±5; NA | $ | NA | NA | NA | NA | NA | Second task force, 1987[59] | NA[3] | 95th percentile | 30 (33.3%) | NA | NA |
| Pieruzzi, 2015[61] | RC | NA | 237 | PreHT 92 (38.8%), HT 145 (61.2%) | NA | 141 (59.5%) | NA[3] | 112 (47.3%) | NA[3] | NA | NA[3] | NA | NA[3] | 4th Report[2], Lurbe, 2009[29] | NA[3] | 95th percentile | 98 (41.4%) | NA | CR 50 (21.1%) CH 53 (22.4%) EH 45 (18.99%) |
| Zamojska, 2015[62] | RC | NA | 34 | NA | 15.3±2.1 | 27 (79.4%) | 21.8±2.05; NA | 0 | NA | 132.47±5.53; NA | 136.6±6.01; NA | NA | 78.38±6.64; NA | Urbina, 2008[9] | 32.19±7.33 | 95th percentile | 3 (8.8%) | NA | NA |
| Lee, 2015[63] | RC | NA | 29 | NA | 15.7±2.0 | NA | 26.3±5.3; NA | NA | NA | NA | NA | NA | NA | NA | 40.7±9.8 | >51g/$m^{2.7}$ | 4 (13.8%) | NA | NA |

Continuous data represented as mean values with standard deviation, unless specified otherwise.

[1] Interquartile range. [2] Data range. [3] Data available only for specific subgroups. [4] N=46. [5] 95% confidence interval. [6] n=100. [7] n=101. * Median. [&] Obese or overweight. [&&] Overweight/obesity defined as ≥85th percentile and reported together. $ Obese patients were excluded.

AmbHT – Ambulatory Hypertension. BMI – body mass index. CC – Community sample. DBP – diastolic blood pressure. n – number of patients. HN – High normal. HT1 – hypertension stage 1. HT2 – hypertension stage 2. LVH – left ventricular hypertrophy. LVMI – left ventricular mass index. NA – not available or not applicable. PH – primary hypertension. PreHT – Prehypertension. RC – referral clinic. SBP – systolic blood pressure. SevAmbHT – severe ambulatory hypertension. SLVH – severe left ventricular hypertrophy. WC – Waist circumference. WCH – white coat hypertension.

**Supplemental Table 4.** Meta-regression (univariable) with explanatory variables for LVH prevalence in the main analysis sample (LVH defined as LVMI >95$^{th}$ percentile)

| Variable | Estimate | 95% CI | P value |
|---|---|---|---|
| Age | -0.007 | -0.098 - 0.084 | 0.89 |
| Male percentage | -0.040 | -0.666 - 0.586 | 0.90 |
| BMI | 0.030 | -0.008 - 0.069 | 0.12 |
| WCz | 0.024 | -0.212 - 0.261 | 0.84 |

BMI, body mass index; CI, confidence interval; WCz, waist circumference z-score.

**Supplemental Figure 1.** Forest plot showing prevalence of LVH in the subset of studies from specialty clinics, LVH defined by ultrasound estimated LVMI ≥95th percentile or 38.6 g/m$^{2.7}$ and excluding subjects with WCH and/or preHT (25 studies, 29 study cohorts)

| Study | Events | Total | Weight | IV, Random, 95% CI |
|---|---|---|---|---|
| Assadi 2007 | 23 | 64 | 3.6% | 0.359 [0.243; 0.489] |
| Litwin 2006 | 30 | 72 | 3.7% | 0.417 [0.302; 0.539] |
| Sorof 2003 | 13 | 32 | 3.0% | 0.406 [0.237; 0.594] |
| Liu 2021 | 12 | 45 | 3.3% | 0.267 [0.146; 0.419] |
| Obrycki (AmbPreHT) 2020 | 5 | 30 | 2.9% | 0.167 [0.056; 0.347] |
| Obrycki (AmbHT) 2020 | 10 | 22 | 2.6% | 0.455 [0.244; 0.678] |
| Obrycki (SevAmbHT) 2020 | 31 | 70 | 3.7% | 0.443 [0.324; 0.567] |
| Litwin 2009 | 28 | 70 | 3.7% | 0.400 [0.285; 0.524] |
| Litwin 2007 | 46 | 113 | 4.0% | 0.407 [0.316; 0.504] |
| Hou 2021 | 8 | 65 | 3.6% | 0.123 [0.055; 0.228] |
| Trojanek 2020 | 17 | 80 | 3.8% | 0.212 [0.129; 0.318] |
| Urbina 2019 | 25 | 91 | 3.8% | 0.275 [0.186; 0.378] |
| Shilly 2019 | 47 | 119 | 4.0% | 0.395 [0.307; 0.489] |
| Litwin (AmbPreHT) 2019 | 3 | 29 | 2.9% | 0.103 [0.022; 0.274] |
| Litwin (AmbHT) 2019 | 8 | 41 | 3.2% | 0.195 [0.088; 0.349] |
| Litwin (SevAmbHT) 2019 | 31 | 97 | 3.9% | 0.320 [0.229; 0.422] |
| Niemirska 2013 | 22 | 50 | 3.4% | 0.440 [0.300; 0.587] |
| Litwin 2010 | 40 | 86 | 3.8% | 0.465 [0.357; 0.576] |
| South 2017 | 60 | 101 | 3.9% | 0.594 [0.492; 0.691] |
| Ramaswamy 2015 | 4 | 24 | 2.7% | 0.167 [0.047; 0.374] |
| Śladowska-Kozłowska 2015 | 39 | 126 | 4.0% | 0.310 [0.230; 0.398] |
| Sorof 2004 | 21 | 43 | 3.3% | 0.488 [0.333; 0.645] |
| Kimball 1993 | 30 | 90 | 3.8% | 0.333 [0.237; 0.441] |
| Niemirska 2016 | 22 | 109 | 3.9% | 0.202 [0.131; 0.289] |
| Litwin 2013 | 4 | 23 | 2.6% | 0.174 [0.050; 0.388] |
| Hacıhamdiog˘lu 2021 | 11 | 44 | 3.3% | 0.250 [0.132; 0.403] |
| Stabouli 2009 | 5 | 25 | 2.7% | 0.200 [0.068; 0.407] |
| Pieruzzi (HT) 2015 | 65 | 145 | 4.1% | 0.448 [0.366; 0.533] |
| Zamojska 2015 | 3 | 34 | 3.0% | 0.088 [0.019; 0.237] |
| **Total (95% CI)** | | **1940** | **100.0%** | **0.314 [0.268; 0.363]** |

Heterogeneity: $Tau^2 = 0.0143$; $Chi^2 = 134.63$, df = 28 ($P < 0.01$); $I^2 = 79\%$

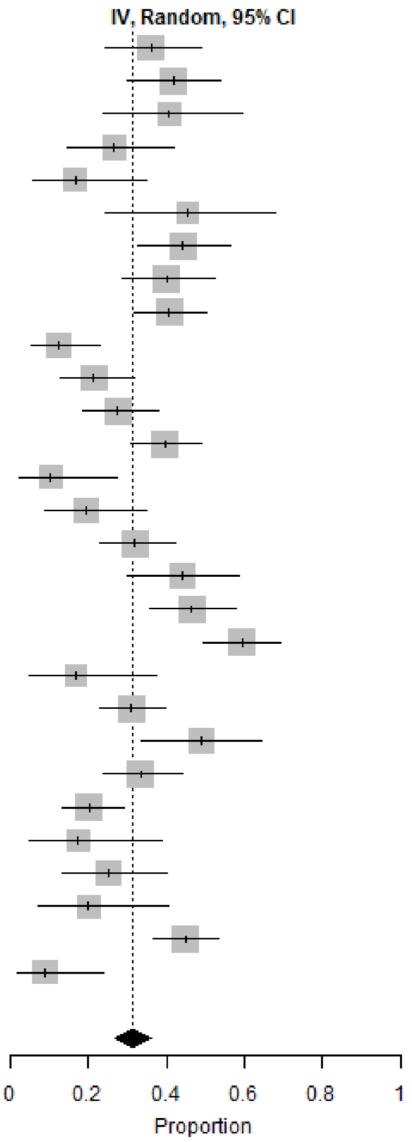

**Supplemental Figure 2.** Forest plot showing the prevalence of severe LVH in studies with available data (12 studies, 15 study cohorts)

| Study | Events | Total | Weight | IV, Random, 95% CI |
|---|---|---|---|---|
| Lande 2006 | 1 | 28 | 5.8% | 0.036 [0.001; 0.183] |
| Litwin 2010 | 10 | 86 | 7.5% | 0.116 [0.057; 0.203] |
| Litwin (HT) 2009 | 9 | 70 | 7.2% | 0.129 [0.061; 0.230] |
| Litwin (WCH) 2009 | 6 | 36 | 6.2% | 0.167 [0.064; 0.328] |
| Litwin 2008 | 14 | 122 | 7.8% | 0.115 [0.064; 0.185] |
| Litwin 2007 | 14 | 113 | 7.7% | 0.124 [0.069; 0.199] |
| Litwin 2006 | 10 | 72 | 7.3% | 0.139 [0.069; 0.241] |
| Niemirska (a) 2013 | 8 | 50 | 6.8% | 0.160 [0.072; 0.291] |
| Obrycki (AmbHT) 2020 | 0 | 22 | 5.3% | 0.000 [0.000; 0.154] |
| Obrycki (AmbPreHT) 2020 | 0 | 30 | 5.9% | 0.000 [0.000; 0.116] |
| Obrycki (SevAmbHT) 2020 | 2 | 70 | 7.2% | 0.029 [0.003; 0.099] |
| Trojanek 2020 | 2 | 80 | 7.4% | 0.025 [0.003; 0.087] |
| Sorof 2002 | 10 | 37 | 6.3% | 0.270 [0.138; 0.441] |
| Yang 2021 | 16 | 28 | 5.8% | 0.571 [0.372; 0.755] |
| Lee 2015 | 4 | 29 | 5.9% | 0.138 [0.039; 0.317] |
| **Total (95% CI)** | | **873** | **100.0%** | **0.109 [0.064; 0.164]** |

Heterogeneity: $Tau^2 = 0.0176$; $Chi^2 = 70.61$, df = 14 (P < 0.01); $I^2 = 80\%$

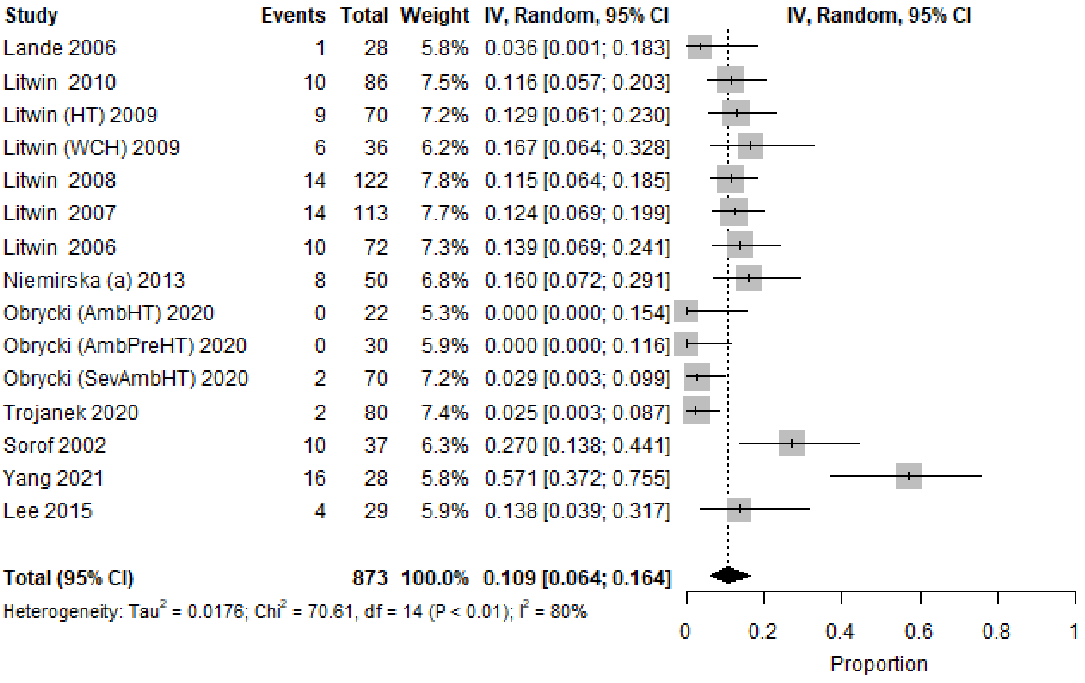

**Supplemental Figure 3.** Forest plot showing prevalence of LVH in the subset of studies with community samples (2 studies)

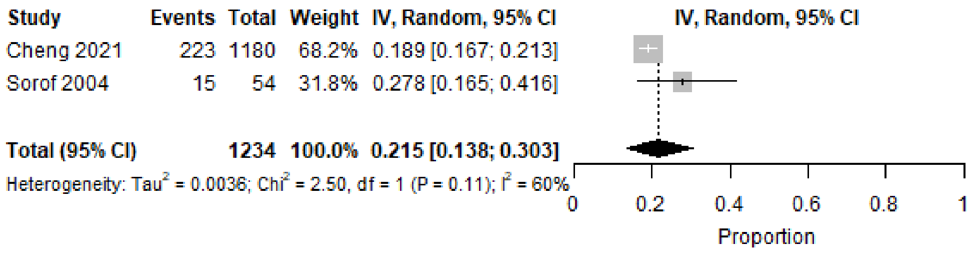


*Note: one study was excluded due due to inclusion of relative wall thickness in the definition of LVH (Meng 2015)*

**Supplemental Figure 4.** Forest plot showing prevalence of LVH in the subset of samples with WCH only (4 study cohorts)

| Study | Events | Total | Weight | IV, Random, 95% CI |
|---|---|---|---|---|
| Litwin 2019 | 27 | 127 | 27.6% | 0.213 [0.145; 0.294] |
| Litwin 2009 | 13 | 36 | 24.7% | 0.361 [0.208; 0.538] |
| Lande 2008 | 0 | 27 | 23.5% | 0.000 [0.000; 0.128] |
| Ramaswamy 2015 | 12 | 32 | 24.2% | 0.375 [0.211; 0.563] |
| **Total (95% CI)** | | **222** | **100.0%** | **0.202 [0.059; 0.398]** |

Heterogeneity: $Tau^2 = 0.0396$; $Chi^2 = 24.74$, df = 3 ($P < 0.01$); $I^2 = 88\%$

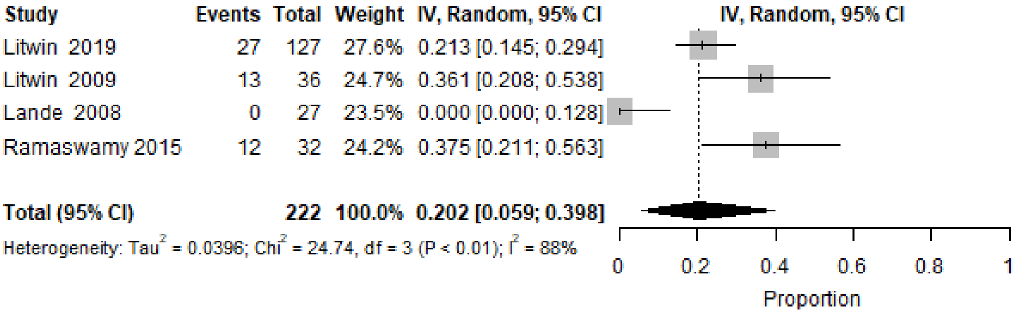


*Note: two studies were excluded as being mixed samples (included community and specialty clinic patients: McNiece 2007, Gupta-Malhotra 2016) and one because MRI was used to define LVH (Sarnecki 2022)*

**Supplemental Figure 5.** Forest plot showing prevalence of LVH in the subset of samples with preHT only (3 study cohorts)

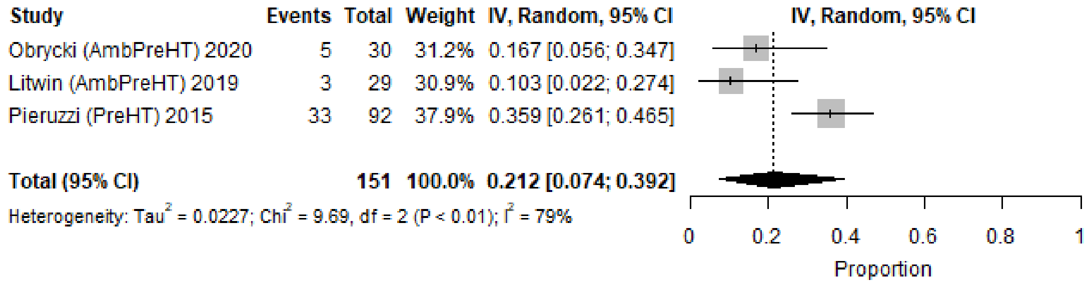


*Note: two studies were excluded for small sample sizes (Stabouli 2009, Ramaswamy 2015)*

**Supplemental Figure 6.** Summary of critical appraisal using Joanna Briggs Institute Critical Appraisal Checklist for Studies Reporting Prevalence Data

| Study | 1. Was the sample frame appropriate to address the target population? | 2. Were study participants sampled in an appropriate way? | 3. Was the sample size adequate? | 4 Were the study subjects and the setting described in detail? | 5. Was the data analysis conducted with sufficient coverage of the | 6. Were valid methods used for the identification of the condition? | 7. Was the condition measured in a standard, reliable way for all participants? | 8. Was there appropriate statistical analysis? | 9. Was the response rate adequate, and if not, was the low response rate managed appropriately? | Overall score | Risk of bias |
|---|---|---|---|---|---|---|---|---|---|---|---|
| Liu Y 2022 | Yes | No | Yes | No | NA | No | Unclear | Yes | Yes | 4 | High |
| Cheng 2021 | Yes | Yes | Yes | No | NA | Yes | Unclear | Yes | No | 5 | Low |
| Hacıhamdioğlu (a) 2021 | Yes | No | No | No | NA | Unclear | Unclear | Yes | No | 2 | High |
| Liu W 2021 | No | No | No | No | NA | No | Unclear | Yes | Unclear | 1 | High |
| Yang 2021 | Yes | No | No | No | NA | No | No | Yes | No | 2 | High |
| Hou M 2021 | Yes | No | No | No | NA | Yes | No | Yes | Yes | 4 | High |
| Liu 2020 | Unclear | No | No | No | NA | No | No | Yes | Yes | 2 | High |
| Trojanek 2020 | Yes | No | No | No | NA | Yes | Unclear | Yes | No | 3 | High |
| Urbina 2019 | No | No | No | No | NA | Yes | Yes | Yes | Yes | 4 | High |
| Antolini 2019 | Yes | Yes | Yes | No | NA | Yes | Unclear | Yes | Yes | 6 | Low |
| Shilly 2019 | Yes | No | No | No | NA | Yes | Unclear | Yes | Yes | 4 | High |
| Litwin (a) 2019 | Yes | No | No | No | NA | Yes | Yes | Yes | Yes | 5 | Low |
| Conkar 2015 | Yes | No | No | No | NA | No | Yes | Yes | Yes | 4 | High |
| Meng 2015 | No | Yes | No | No | NA | No | Unclear | Yes | No | 2 | High |
| Agu 2014 | No | No | No | No | NA | No | Yes | Yes | Yes | 3 | High |
| Niemirska (a) 2013 | No | No | No | No | NA | Yes | Unclear | Yes | Unclear | 2 | High |
| Sharma 2013 | Yes | No | No | No | NA | Yes | Unclear | Yes | Unclear | 3 | High |
| Litwin (b) 2010 | Yes | No | No | Yes | NA | Yes | Yes | Yes | Unclear | 5 | Low |
| Richey PA 2010 | Yes | No | No | Yes | NA | No | Yes | Yes | Unclear | 4 | High |
| Litwin (c) 2009 | Yes | No | No | Yes | NA | No | Unclear | No | No | 2 | High |
| Stabouli S 2009 | Yes | No | No | Yes | NA | Yes | Yes | Yes | Unclear | 5 | Low |
| Lande MB 2008 | Yes | No | No | Yes | NA | No | Unclear | Yes | No | 3 | High |
| Litwin (d) 2008 | Yes | No | No | Yes | NA | No | Unclear | Yes | No | 3 | High |
| Brady TM 2008 | Yes | No | No | No | NA | No | No | Yes | Yes | 3 | High |
| McNiece KL 2007 | Unclear | No | No | Yes | NA | No | Unclear | No | Unclear | 1 | High |
| Litwin (e) 2007 | Unclear | No | No | Yes | NA | No | Unclear | Yes | No | 2 | High |
| Assadi (b) 2007 | Unclear | No | No | Yes | NA | No | Unclear | Yes | Yes | 3 | High |
| South 2017 | Yes | No | No | No | NA | Yes | Unclear | Yes | Yes | 4 | High |
| Gupta-Malhotra (b) 2016 | No | No | No | Yes | NA | No | Yes | Yes | Yes | 4 | High |
| Ramaswamy 2015 | Yes | No | No | Yes | NA | Unclear | Yes | Yes | No | 4 | High |
| Śladowska-Kozłowska (a) 2015 | Yes | No | No | No | NA | Yes | Unclear | Yes | No | 3 | High |
| Lande 2006 | Yes | No | No | Yes | NA | Yes | Yes | Yes | Yes | 6 | Low |
| Litwin (f) 2006 | Yes | No | No | No | NA | No | Unclear | Yes | Yes | 3 | High |
| Sorof (a) 2004 | Yes | Yes | No | No | NA | Unclear | Yes | Yes | Yes | 5 | Low |
| Kimball 1993 | No | No | No | No | NA | Unclear | Unclear | Yes | Yes | 2 | High |
| Niemirska (b) 2016 | Yes | No | no | no | NA | Yes | Unclear | Yes | Yes | 4 | High |
| Litwin (g) 2013 | Yes | no | no | no | NA | Yes | Unclear | Yes | No | 3 | High |
| Flynn 2005 | Yes | No | No | Yes | NA | No | No | Yes | Yes | 4 | High |
| Sorof (b) 2003 | Yes | No | No | No | NA | No | Unclear | Yes | Yes | 3 | High |
| Belsha 1998 | No | No | No | Yes | NA | No | Unclear | Yes | No | 2 | High |
| Niemirska (c) 2014 | Yes | No | No | No | NA | Yes | Yes | Yes | Yes | 5 | Low |
| Obrycki 2021 | No | No | No | Yes | NA | Yes | No | Yes | Yes | 4 | High |
| Sarnecki 2022 | No | No | No | No | NA | No | No | Yes | No | 1 | High |
| Sorof (c) 2002 | Unclear | No | No | No | NA | No | Unclear | Yes | Unclear | 1 | High |
| Pieruzzi 2015 | Yes | No | No | Yes | NA | Yes | No | Yes | Unclear | 4 | High |
| Zamojska 2015 | Yes | No | No | Yes | NA | Yes | No | Yes | Yes | 5 | Low |
| Lee 2015 | No | No | No | No | NA | No | No | Yes | Yes | 2 | High |

**Supplemental Figure 7.** Funnel plot for the analysis of the subset of studies from specialty clinics, LVH defined by ultrasound estimated LVMI ≥95th percentile and excluding subjects with WCH and/or preHT

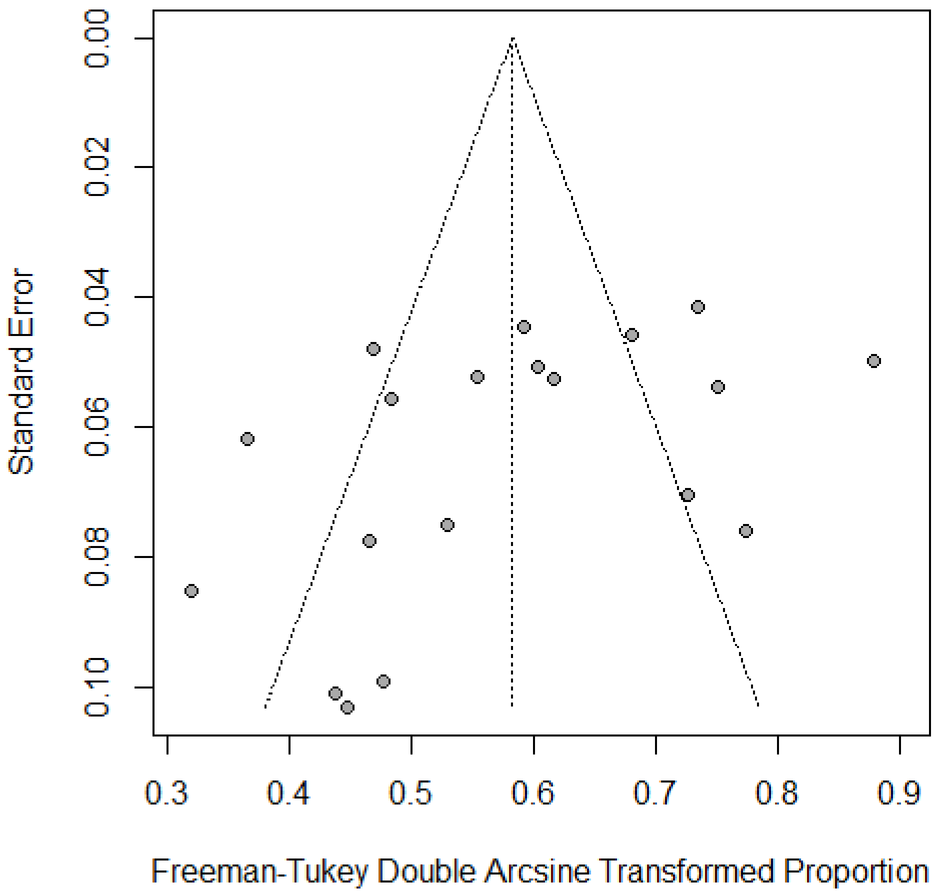

**Supplemental Figure 8.** Funnel plot for the analysis of the subset of studies from specialty clinics, LVH defined by ultrasound estimated LVMI ≥95th percentile or 38.6 $g/m^{2.7}$ and excluding subjects with WCH and/or preHT

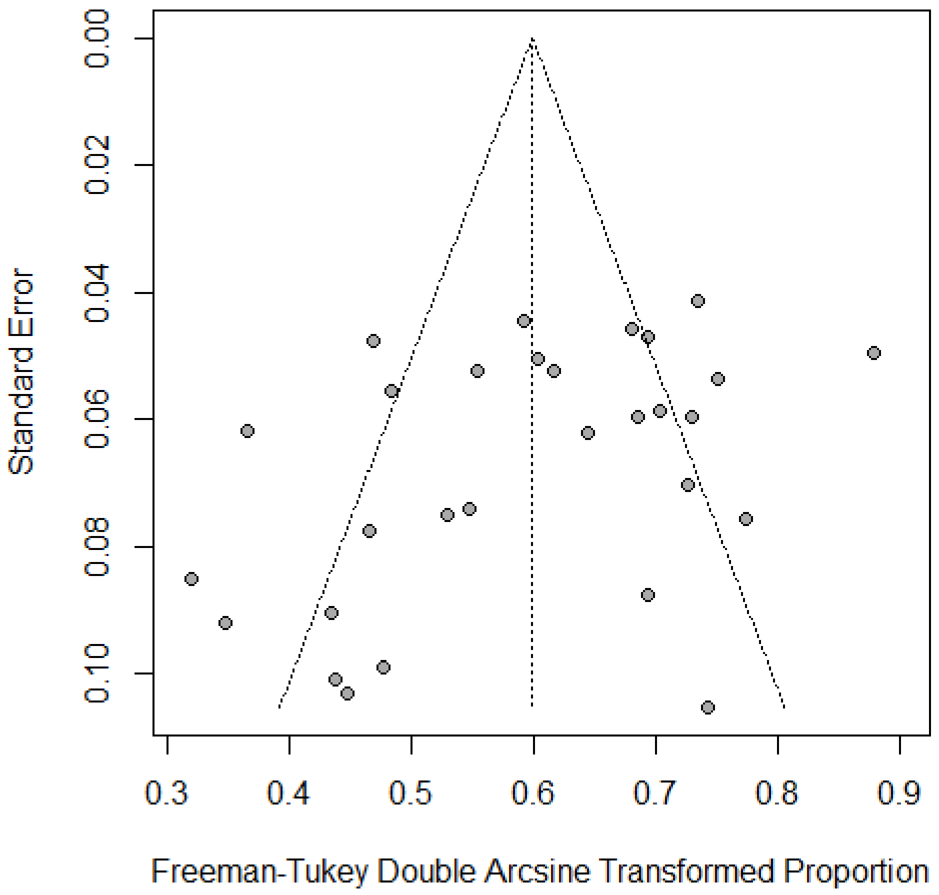

## References


1. Liu Y, Shi L, Lin Y, Zhang M, Chen F, Li A, et al. Relationship between serum 25-hydroxyvitamin D and target organ damage in children with essential hypertension. Journal of Human Hypertension 2021. 2022 Jan 9;1–6. Available from: https://www.nature.com/articles/s41371-021-00622-4
2. Falkner B, Daniels SR, Flynn JT, Gidding S, Green LA, Ingelfinger JR et al. The fourth report on the diagnosis, evaluation, and treatment of high blood pressure in children and adolescents. Pediatrics. 2004 Aug;114(2 III):555-576. https://doi.org/10.1542/peds.114.2.S2.555
3. Sarnecki J, Obrycki Ł, Feber J, Chełstowska S, Jurkiewicz E, Litwin M. Isolated systolic hypertension is associated with increased left ventricular mass index and aortic stiffness in adolescents: a cardiac magnetic resonance study. J Hypertens. 2022 May 1;40(5):985–95. Available from: https://pubmed.ncbi.nlm.nih.gov/35191414/
4. Lurbe E, Agabiti-Rosei E, Cruickshank JK, Dominiczak A, Erdine S, Hirth A, et al. 2016 European Society of Hypertension guidelines for the management of high blood pressure in children and adolescents. J Hypertens. 2016;34(10):1887–920. Available from: https://pubmed.ncbi.nlm.nih.gov/27467768/
5. Flynn JT, Daniels SR, Hayman LL, Maahs DM, McCrindle BW, Mitsnefes M, et al. Update: ambulatory blood pressure monitoring in children and adolescents: a scientific statement from the American Heart Association. Hypertension. 2014;63(5):1116–35. Available from: https://pubmed.ncbi.nlm.nih.gov/24591341/
6. Cheng H, Xi B, Liu J, Yan Y, Mi J. Performance of different adiposity measures for predicting left ventricular remodeling in Chinese hypertensive youth. Scientific Reports 2021 11:1. 2021 Nov 9;11(1):1–6. Available from: https://www.nature.com/articles/s41598-021-00978-0
7. Flynn JT, Kaelber DC, Baker-Smith CM, Blowey D, Carroll AE, Daniels SR, et al. Clinical Practice Guideline for Screening and Management of High Blood Pressure in Children and Adolescents. Pediatrics. 2017 Sep 1;140(3). Available from: https://pubmed.ncbi.nlm.nih.gov/28827377/
8. Hacıhamdioğlu D, Koçak G, Doğan BN, Koyuncu E. Challenges in choosing the appropriate guidelines for use in children and adolescents with hypertension. Archives de pediatrie: organe officiel de la Societe francaise de pediatrie. 2021 Aug 1;28(6):451–8. Available from: https://pubmed.ncbi.nlm.nih.gov/34226065/
9. Urbina E, Alpert B, Flynn J, Hayman L, Harshfield GA, Jacobson M, et al. Ambulatory blood pressure monitoring in children and adolescents: Recommendations for standard assessment: A scientific statement from the american heart association atherosclerosis, hypertension, and obesity in youth committee of the council on cardiovascular disease in the young and the council for high blood pressure research. Hypertension. 2008 Sep 1;52(3):433–51.
10. Liu W, Hou C, Hou M, Xu QQ, Wang H, Gu PP, et al. Ultrasonography to detect cardiovascular damage in children with essential hypertension. Cardiovascular Ultrasound. 2021 Dec 1;19(1). Available from: /pmc/articles/PMC8296659/
11. Fan, H., Yan, Y. K., & Mi, J. (2017). Updating blood pressure references for Chinese children aged 3-17 years. *Chin J Hypertens, 25*(5), 428-435.
12. Yang Z, Huang Y, Qin Y, Pang Y. Clinical Characteristics and Factors Associated With Hypertension in 205 Hospitalized Children: A Single-Center Study in Southwest China. Front Pediatr. 2021 Apr 7;9. Available from: https://pubmed.ncbi.nlm.nih.gov/33898356/

13. Hou M, Cao L, Ding Y, Chen Y, Wang B, Shen J, et al. Neutrophil to Lymphocyte Ratio Is Increased and Associated With Left Ventricular Diastolic Function in Newly Diagnosed Essential Hypertension Children. Frontiers in Pediatrics. 2021 May 19;9:576005. Available from: /pmc/articles/PMC8169980/
14. Dong Y, Ma J, Song Y, Dong B, Wang Z, Yang Z, et al. National Blood Pressure Reference for Chinese Han Children and Adolescents Aged 7 to 17 Years. Hypertension. 2017 Nov 1;70(5):897–906. Available from: https://www.ahajournals.org/doi/abs/10.1161/HYPERTENSIONAHA.117.09983
15. Liu Y, Lin Y, Zhang MM, Li XH, Liu YY, Zhao J, et al. The relationship of plasma renin, angiotensin, and aldosterone levels to blood pressure variability and target organ damage in children with essential hypertension. BMC Cardiovasc Disord. 2020 Jun 16;20(1). Available from: https://pubmed.ncbi.nlm.nih.gov/32546130/
16. Trojanek JB, Niemirska A, Grzywa R, Wierzbicka A, Obrycki Ł, Kułaga Z, et al. Leukocyte matrix metalloproteinase and tissue inhibitor gene expression patterns in children with primary hypertension. J Hum Hypertens. 2020 May 1;34(5):355–63. Available from: https://pubmed.ncbi.nlm.nih.gov/30926902/
17. Obrycki Ł, Feber J, Derezinski T, Lewandowska W, Kulaga Z, Litwin M. Hemodynamic Patterns and Target Organ Damage in Adolescents With Ambulatory Prehypertension. Hypertension. 2020;75(3):826–34. Available from: https://pubmed.ncbi.nlm.nih.gov/31884853/
18. Urbina EM, Mendizábal B, Becker RC, Daniels SR, Falkner BE, Hamdani G, et al. Association of Blood Pressure Level With Left Ventricular Mass in Adolescents. Hypertension. 2019 Sep 1;74(3):590–6. Available from: https://pubmed.ncbi.nlm.nih.gov/31327264/
19. Antolini L, Giussani M, Orlando A, Nava E, Valsecchi MG, Parati G, et al. Nomograms to identify elevated blood pressure values and left ventricular hypertrophy in a paediatric population: American Academy of Pediatrics Clinical Practice vs. Fourth Report/European Society of Hypertension Guidelines. J Hypertens. 2019 Jun 1;37(6):1213–22. Available from: https://pubmed.ncbi.nlm.nih.gov/31022109/
20. Shilly S, Merchant K, Singer P, Frank R, Gurusinghe S, Infante L, et al. Left ventricular cardiac geometry and ambulatory blood pressure in children. The Journal of Clinical Hypertension. 2019 May 1;21(5):566. Available from: /pmc/articles/PMC8030399/
21. Litwin M, Obrycki Ł, Niemirska A, Sarnecki J, Kułaga Z. Central systolic blood pressure and central pulse pressure predict left ventricular hypertrophy in hypertensive children. Pediatr Nephrol. 2019 Apr 1;34(4):703–12. Available from: https://pubmed.ncbi.nlm.nih.gov/30426220/
22. South AM, Arguelles L, Finer G, Langman CB. Race, obesity, and the renin-angiotensin-aldosterone system: treatment response in children with primary hypertension. Pediatr Nephrol. 2017 Sep 1;32(9):1585–94. Available from: https://pubmed.ncbi.nlm.nih.gov/28411317/
23. Chobanian A v., Bakris GL, Black HR, Cushman WC, Green LA, Izzo JL, et al. Seventh report of the Joint National Committee on Prevention, Detection, Evaluation, and Treatment of High Blood Pressure. Hypertension. 2003 Dec;42(6):1206–52. Available from: https://pubmed.ncbi.nlm.nih.gov/14656957/
24. Gupta-Malhotra M, Hamzeh RK, Poffenbarger T, McNiece-Redwine K, Hashmi SS. Myocardial Performance Index in Childhood Onset Essential Hypertension and White Coat Hypertension. Am J Hypertens. 2016 Mar 1;29(3):379–87. Available from: https://pubmed.ncbi.nlm.nih.gov/26271107/

25. Niemirska A, Litwin M, Trojanek J, Gackowska L, Kubiszewska I, Wierzbicka A, et al. Altered matrix metalloproteinase 9 and tissue inhibitor of metalloproteinases 1 levels in children with primary hypertension. J Hypertens. 2016 Sep 1;34(9):1815–22. Available from: https://pubmed.ncbi.nlm.nih.gov/27379542/
26. Ramaswamy P, Chikkabyrappa S, Donda K, Osmolovsky M, Rojas M, Rafii D. Relationship of ambulatory blood pressure and body mass index to left ventricular mass index in pediatric patients with casual hypertension. J Am Soc Hypertens. 2016 Feb 1;10(2):108–14. Available from: https://pubmed.ncbi.nlm.nih.gov/26725015/
27. Śladowska-Kozłowska J, Litwin M, Niemirska A, Wierzbicka A, Roszczynko M, Szperl M. Associations of the eNOS G894T gene polymorphism with target organ damage in children with newly diagnosed primary hypertension. Pediatric Nephrology. 2015 Dec 1;30(12):2189–97. Available from: https://link.springer.com/article/10.1007/s00467-015-3164-9
28. Conkar S, Yilmaz E, Hacikara Ş, Bozabali S, Mir S. Is Daytime Systolic Load an Important Risk Factor for Target Organ Damage in Pediatric Hypertension? J Clin Hypertens (Greenwich). 2015 Oct 1;17(10):760–6. Available from: https://pubmed.ncbi.nlm.nih.gov/26140344/
29. Lurbe E, Cifkova R, Cruickshank JK, Dillon MJ, Ferreira I, Invitti C, et al. Management of high blood pressure in children and adolescents: recommendations of the European Society of Hypertension. J Hypertens. 2009;27(9):1719–42. Available from: https://pubmed.ncbi.nlm.nih.gov/19625970/
30. Meng L, Hou D, Zhao X, Hu Y, Liang Y, Liu J, et al. Cardiovascular target organ damage could have been detected in sustained pediatric hypertension. Blood Press. 2015 Sep 3;24(5):284–92. Available from: https://pubmed.ncbi.nlm.nih.gov/26024395/
31. Mi J, Wang TY, Meng LH, Zhu GJ, Han SM, Zhong Y, et al. Development of blood pressure reference standards for Chinese children and adolescents. Chin J Evid Based Pediatr. 2010;5:4–14.
32. Niemirska A, Obrycki Ł, Litwin M. Utility of pulse wave velocity and pulse wave analysis in assessment of hypertensive target organ damage in children with primary hypertension. Arterial Hypertension}. 194 - 203 (2014), https://journals.viamedica.pl/arterial_hypertension/article/view/41581
33. Agu NC, McNiece Redwine K, Bell C, Garcia KM, Martin DS, Poffenbarger TS, et al. Detection of Early Diastolic Alterations by Tissue Doppler Imaging in Untreated Childhood-Onset Essential Hypertension. J Am Soc Hypertens. 2014;8(5):303. Available from: /pmc/articles/PMC4034523/
34. Litwin M, Michałkiewicz J, Trojanek J, Niemirska A, Wierzbicka A, Szalecki M. Altered genes profile of renin-angiotensin system, immune system, and adipokines receptors in leukocytes of children with primary hypertension. Hypertension. 2013 Feb;61(2):431–6. Available from: https://pubmed.ncbi.nlm.nih.gov/23266543/
35. Niemirska A, Litwin M, Feber J, Jurkiewicz E. Blood pressure rhythmicity and visceral fat in children with hypertension. Hypertension. 2013;62(4):782–8. Available from: https://pubmed.ncbi.nlm.nih.gov/23959553/
36. Sharma AP, Mohammed J, Thomas B, Lansdell N, Norozi K, Filler G. Nighttime blood pressure, systolic blood pressure variability, and left ventricular mass index in children with hypertension. Pediatr Nephrol. 2013 Aug;28(8):1275–82. Available from: https://pubmed.ncbi.nlm.nih.gov/23564040/

37. Litwin M, Niemirska A, Śladowska-Kozlowska J, Wierzbicka A, Janas R, Wawer ZT, et al. Regression of target organ damage in children and adolescents with primary hypertension. Pediatr Nephrol. 2010 Dec;25(12):2489–99. Available from: https://pubmed.ncbi.nlm.nih.gov/20730452/
38. Richey PA, Disessa TG, Somes GW, Alpert BS, Jones DP. Left ventricular geometry in children and adolescents with primary hypertension. Am J Hypertens. 2010 Jan;23(1):24–9. Available from: https://pubmed.ncbi.nlm.nih.gov/19851297/
39. Lurbe E, Sorof JM, Daniels SR. Clinical and research aspects of ambulatory blood pressure monitoring in children. J Pediatr. 2004;144(1):7–16. Available from: https://pubmed.ncbi.nlm.nih.gov/14722512/
40. Litwin M, Niemirska A, Ruzicka M, Feber J. White coat hypertension in children: not rare and not benign? J Am Soc Hypertens. 2009 Nov;3(6):416–23. Available from: https://pubmed.ncbi.nlm.nih.gov/20409984/
41. Stabouli S, Kotsis V, Rizos Z, Toumanidis S, Karagianni C, Constantopoulos A, et al. Left ventricular mass in normotensive, prehypertensive and hypertensive children and adolescents. Pediatr Nephrol. 2009;24(8):1545–51. Available from: https://pubmed.ncbi.nlm.nih.gov/19444486/
42. Wuhl E, Witte K, Soergel M, Mehls O, Schaefer F, German Working Group on Pediatric Hypertension (2002) Distribution of 24-h ambulatory blood pressure in children: normalized reference values and role of body dimensions. J Hypertens 20:1995–2007
43. Lande MB, Meagher CC, Fisher SG, Belani P, Wang H, Rashid M. Left ventricular mass index in children with white coat hypertension. J Pediatr. 2008 Jul;153(1):50. Available from: /pmc/articles/PMC2516747/
44. Litwin M, Śladowska J, Syczewska M, Niemirska A, Daszkowska J, Antoniewicz J, et al. Different BMI cardiovascular risk thresholds as markers of organ damage and metabolic syndrome in primary hypertension. Pediatr Nephrol. 2008 May;23(5):787–96. Available from: https://pubmed.ncbi.nlm.nih.gov/18253758/
45. Brady TM, Fivush B, Flynn JT, Parekh R. Ability of blood pressure to predict left ventricular hypertrophy in children with primary hypertension. J Pediatr. 2008;152(1). Available from: https://pubmed.ncbi.nlm.nih.gov/18154904/
46. Flynn JT. Evaluation and management of hypertension in childhood. Progress in Pediatric Cardiology. 2001 Jan 1;12(2):177–88.
47. McNiece KL, Gupta-Malhotra M, Samuels J, Bell C, Garcia K, Poffenbarger T, et al. Left ventricular hypertrophy in hypertensive adolescents: analysis of risk by 2004 National High Blood Pressure Education Program Working Group staging criteria. Hypertension. 2007 Aug;50(2):392–5. Available from: https://pubmed.ncbi.nlm.nih.gov/17592068/
48. Soergel M, Kirschstein M, Busch C, Danne T, Gellermann J, Holl R, Krull F, Reichert H, Reusz GS, Rascher W. Oscillometric twenty-four-hour ambulatory blood pressure values in healthy children and adolescents: a multicenter trial including 1141 subjects. J Pediatr. 1997;130:178 –184.
49. Litwin M, Śladowska J, Antoniewicz J, Niemirska A, Wierzbicka A, Daszkowska J, et al. Metabolic abnormalities, insulin resistance, and metabolic syndrome in children with primary hypertension. Am J Hypertens. 2007 Aug;20(8):875–82. Available from: https://pubmed.ncbi.nlm.nih.gov/17679036/

50. Assadi F. Effect of microalbuminuria lowering on regression of left ventricular hypertrophy in children and adolescents with essential hypertension. Pediatr Cardiol. 2007 Feb;28(1):27–33. Available from: https://pubmed.ncbi.nlm.nih.gov/17308944/
51. Lande MB, Carson NL, Roy J, Meagher CC. Effects of childhood primary hypertension on carotid intima media thickness: a matched controlled study. Hypertension. 2006 Jul;48(1):40–4. Available from: https://pubmed.ncbi.nlm.nih.gov/16735644/
52. Litwin M, Niemirska A, Śladowska J, Antoniewiez J, Daszkowska J, Wierzbicka A, et al. Left ventricular hypertrophy and arterial wall thickening in children with essential hypertension. Pediatr Nephrol. 2006 Jun;21(6):811–9. Available from: https://pubmed.ncbi.nlm.nih.gov/16565870/
53. Flynn JT, Alderman MH. Characteristics of children with primary hypertension seen at a referral center. Pediatr Nephrol. 2005 Jul;20(7):961–6. Available from: https://pubmed.ncbi.nlm.nih.gov/15864653/
54. Adolescents NHBPEPWG on HC in C and. Update on the 1987 Task Force Report on High Blood Pressure in Children and Adolescents: A Working Group Report from the National High Blood Pressure Education Program. Pediatrics. 1996 Oct 1;98(4):649–58. Available from: /pediatrics/article/98/4/649/60522/Update-on-the-1987-Task-Force-Report-on-High-Blood
55. Sorof JM, Turner J, Martin DS, Garcia K, Garami Z, Alexandrov A v., et al. Cardiovascular risk factors and sequelae in hypertensive children identified by referral versus school-based screening. Hypertension. 2004 Feb;43(2):214–8. Available from: https://pubmed.ncbi.nlm.nih.gov/14744920/
56. Sorof JM, Alexandrov A v., Cardwell G, Portman RJ. Carotid artery intimal-medial thickness and left ventricular hypertrophy in children with elevated blood pressure. Pediatrics. 2003 Jan 1;111(1):61–6. Available from: https://pubmed.ncbi.nlm.nih.gov/12509555/
57. Sorof JM, Cardwell G, Franco K, Portman RJ. Ambulatory blood pressure and left ventricular mass index in hypertensive children. Hypertension. 2002;39(4):903–8. Available from: https://pubmed.ncbi.nlm.nih.gov/11967247/
58. Influence of Diurnal Blood Pressure Variations on Target Organ Abnormalities in Adolescents With Mild Essential Hypertension. Available from: https://www.infona.pl/resource/bwmeta1.element.elsevier-8e56aabc-7b66-3eb8-b5ab-3dac0e5a5b07
59. Children TF on BPC in. Report of the Second Task Force on Blood Pressure Control in Children—1987. Pediatrics. 1987 Jan 1;79(1):1–25. Available from: /pediatrics/article/79/1/1/54239/Report-of-the-Second-Task-Force-on-Blood-Pressure
60. Kimball TR, Daniels SR, Loggie JMH, Khoury P, Meyer RA. Relation of left ventricular mass, preload, afterload and contractility in pediatric patients with essential hypertension. J Am Coll Cardiol. 1993 Mar 15;21(4):997–1001. Available from: https://pubmed.ncbi.nlm.nih.gov/8450171/
61. Pieruzzi F, Antolini L, Salerno FR, Giussani M, Brambilla P, Galbiati S, Mastriani S, Rebora P, Stella A, Valsecchi MG, Genovesi S. The role of blood pressure, body weight and fat distribution on left ventricular mass, diastolic function and cardiac geometry in children. J Hypertens. 2015 Jun;33(6):1182-92. doi: 10.1097/HJH.0000000000000552.
62. Zamojska J, Niewiadomska-Jarosik K, Wosiak A, Lipiec P, Stańczyk J. Myocardial dysfunction measured by tissue Doppler echocardiography in children with primary arterial hypertension. Kardiol Pol. 2015;73(3):194-200. doi: 10.5603/KP.a2014.0189. Epub 2014 Oct 9. PMID: 25299400.

63. Lee H, Kong YH, Kim KH, Huh J, Kang IS, Song J. Left ventricular hypertrophy and diastolic function in children and adolescents with essential hypertension. Clin Hypertens. 2015 Oct 22;21:21. doi: 10.1186/s40885-015-0031-8. PMID: 26893931; PMCID: PMC4750793.
